# Combined Use of Electroencephalography and Transcranial Electrical Stimulation: A Systematic Review

**DOI:** 10.3390/s25185773

**Published:** 2025-09-16

**Authors:** Pasquale Arpaia, Anna Della Calce, Lucrezia Di Marino, Luciana Lorenzon, Luigi Maffei, Nicola Moccaldi, Pedro M. Ramos

**Affiliations:** 1 Department of Electrical Engineering and Information Technology, University of Naples Federico II, 80125 Naples, Italy; anna.dellacalce2@unina.it (A.D.C.); lucrezia.dimarino2@unina.it (L.D.M.); luigimaffei@live.it (L.M.); nicola.moccaldi@unina.it (N.M.); 2 Interdepartmental Center for Research on Management and Innovation in Healthcare (CIRMIS), University of Naples Federico II, 80125 Naples, Italy; 3 Centro Neurologico Neuroagain, Via Pasquale Biondi, 12, 82016 Montesarchio, Italy; luciana.cinb.it@gmail.com; 4 Instituto de Telecomunicações, Instituto Superior Técnico (IST), 1049-001 Lisboa, Portugal; pedro.m.ramos@tecnico.ulisboa.pt

**Keywords:** EEG, tES, personalization, closed loop

## Abstract

This systematic review examines the combined use of electroencephalography (EEG) and transcranial electrical stimulation (tES) in both clinical and healthy populations. The review focuses on EEG’s role in guiding, monitoring, and evaluating tES interventions and assesses the generalizability of EEG responses to different tES protocols. A comprehensive search across Google Scholar, PubMed, Scopus, IEEE Xplore, ScienceDirect, and Web of Science identified 162 relevant studies using the query: “EEG AND (tDCS OR transcranial direct current stimulation OR tACS OR transcranial alternating current stimulation OR tRNS OR transcranial random noise stimulation OR tPCS OR transcranial pulsed current stimulation)”. Quality was assessed using the Quality Assessment Tool for Quantitative Studies (QATQS). Most studies used EEG post tES to assess neuromodulatory effects, with fewer studies using EEG for protocol design or incorporating real-time EEG for adaptive stimulation. Some studies integrated EEG both before and after stimulation, but considerable heterogeneity in tES parameters and EEG metrics limited reproducibility and comparability. Many studies reported non-significant EEG changes despite standardized approaches. Methodological quality was generally low, and the link between EEG changes and clinical outcomes remains unclear. The findings underscore the potential of EEG-informed, personalized tES protocols, though the use of real-time closed-loop systems remains a limited approach in current research.

## 1. Introduction

Transcranial Electrical Stimulation (tES) is a non-invasive neuromodulation technique able to deliver low-intensity electric currents (<4 mA) to the scalp [1]. TES is applied by placing two or more electrodes on the target area to be stimulated. The delivered currents interact with the membrane potentials of neuronal cells, inducing multilayer effects on the brain and its related functions [2].

Generally, tES techniques are classified based on two main approaches: (i) *physical* approaches, referring to stimulation parameters such as waveform shape (e.g., direct or alternating current), amplitude, electrode montage, and timing of application, and (ii) *intended use* approaches, including hypothesized mechanisms of action (e.g., excitability modulation, network synchronization, or functional connectivity changes), anatomical targets (e.g., transorbital or deep targets), and expected outcomes (e.g., neurorehabilitation). For instance, according to a physical approach, tDCS and tACS are distinguished by the type of applied current—direct or alternating, respectively. In contrast, according to the intended use approach, *trigeminal nerve stimulation* is differentiated from *transorbital stimulation* based on the specific anatomical target target [2,3].

In recent years, there has been a marked increase in interest in tES as a therapeutic intervention for neurological and psychiatric disorders such as epilepsy, Alzheimer’s disease, depression, and chronic pain, among others. These applications are mainly guided by evidence-based recommendations as outlined in the comprehensive guidelines provided by Lefaucheur et al. (2017) [4] and Antal et al. (2017) [5]. The former guidelines evaluate the efficacy of the most common tES technique—namely, transcranial direct current stimulation (tDCS)—in various neurological conditions, offering structured protocols for its clinical use, while the latter describes the application of different tES treatment supported by safety, ethical, and regulatory guidelines.

However, they propose fixed stimulation setups, disregarding the specific characteristics or pathophysiological profiles of individual patients. This lack of personalization contrasts with ongoing research on precision and adaptive medicine, aiming to tailor treatments based on the unique characteristics of each subject.

In this context, electroencephalography (EEG) has been proposed as a promising tool to guide treatment towards more precise and customized approaches. EEG offers a non-invasive assessment of brain activities, allowing for the identification of EEG features associated with specific pathologies and the monitoring of changes induced by tES treatment [6]. The integration of EEG with tES techniques could be a significant step in adapting stimulation treatments to individual needs, thereby enhancing therapeutic effects.

Several reviews have examined the efficacy and methodological aspects of combining EEG with transcranial electrical stimulation (tES). In particular, Choi et al. (2020) [7] and Ruffini et al. (2020) [8] emphasized the potential of EEG-based biomarkers in predicting and monitoring treatment outcomes. Choi et al. provided a systematic overview of closed-loop feedback approaches for sleep analysis, highlighting the role of EEG features in guiding and adapting stimulation protocols. Ruffini et al. discussed broader clinical applications and theoretical frameworks, without focusing on a systematic analysis of experimental studies measuring EEG responses to tES. Beumer et al. (2022) [9] presented a personalized tDCS workflow for epilepsy that integrates imaging and EEG data for segmentation, source localization, and montage optimization. Their contribution targets a single stimulation modality (tDCS) and pathology (epilepsy) but does not include systematic information on stimulation parameters or control conditions, which limits the interpretation of clinical outcomes. Similarly, Simula et al. (2022) [10] reviewed the role of tDCS and tACS in epilepsy, providing an overview of applications but restricting their scope to specific stimulation types, without addressing variability in electrode montages, parameter settings, or study designs. Yang et al. (2021) [11] offered a systematic overview of tES modalities and stimulation parameters in relation to EEG and fNIRS features across multiple disorders. However, their analysis was limited to a subset of clinical populations and excluded studies conducted on healthy participants or those investigating baseline EEG activity as a reference.

Overall, existing reviews have not provided a comprehensive synthesis of the literature based on the PICOT (Population, Intervention, Comparison, Outcome, and Time) framework [12]. Across studies, populations typically include both clinical groups (e.g., patients with epilepsy, depression, or cognitive impairment) and healthy controls. Interventions encompass different forms of tES (tDCS, tACS, and HD-tDCS), often applied either in open-loop or closed-loop paradigms. Comparison is generally established through sham stimulation, alternative montages, or pre–post treatment designs, though some reviews lack a systematic discussion of control strategies. The outcomes most frequently assessed involve EEG-derived features, such as spectral power, connectivity measures, or biomarkers predictive of clinical response, with clinical endpoints evaluated more sporadically. Finally, the time frame varies considerably, with most studies focusing on acute or short-term EEG changes and only a few investigating longitudinal effects or sustained clinical improvements.

In this context, the present review examines scientific contributions employing EEG to guide and monitor tES interventions in both clinical and healthy populations, with a focus on adapting stimulation protocols to individual neurophysiological profiles.

In particular, this review is structured around the following Research Questions (RQs):

- (RQ-I): Is tES guided by EEG data?
- (RQ-II): Is EEG also used to guide tES in real time?
- (RQ-III): Are treatment outcomes assessed through EEG analysis?
- (RQ-IV): Do electroencephalographic outcomes of specific tES protocols generalize across individuals?

## 2. Materials and Methods

### 2.1. Search Strategy

**Figure 1:**
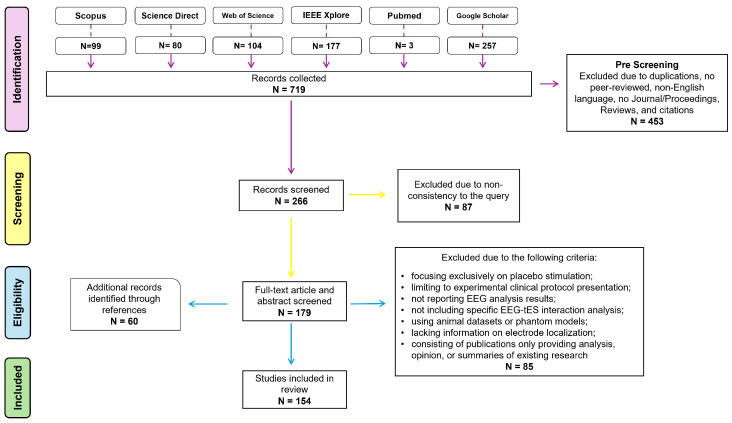
PRISMA flow of article selection process.

The present study was conducted in accordance with the PRISMA guidelines [13], incorporating the recommendations outlined in Kitchenham’s guide [14]. A flow diagram of the database search process is presented in Figure 1, outlining the phases of identification, screening, eligibility, and inclusion. No review protocol was registered for this systematic review. Articles were collected from the Google Scholar, PubMed, Scopus, IEEEXplore, ScienceDirect, and Web of Sciences databases by using the query “EEG AND (TDCS OR transcranial direct current stimulation OR TACS OR transcranial alternating current stimulation OR TRNS OR transcranial random noise stimulation OR TPCS OR transcranial pulsed current stimulation)”, with a restriction to article title [15]. Data from the included studies were charted using a form developed by the research team and pilot-tested on a sample of five studies to ensure consistency and clarity. Article selection and quality evaluation were conducted by the second and third authors: the second author performed the initial evaluation following the QATQS protocol guidelines, and the third author independently repeated the evaluation. Any discrepancies in data charting or study quality assessment were discussed and resolved through consensus, with the involvement of all authors when necessary to reach convergence. Only peer-reviewed papers published in journals or conference proceedings and written in English were included. No date restrictions were applied, and the literature search was conducted through January 2025. Subsequently, the screening process was carried out by combining the results from each source and excluding all duplicates and citations. Titles were manually screened to exclude papers deemed irrelevant or inconsistent with the query. Finally, during the eligibility phase, all remaining full-text papers and abstracts were screened based on the criteria outlined in the following section. The remaining papers in this final phase were included in the review analysis to address the research questions.

### 2.2. Exclusion Criteria

All articles underwent a thorough screening process and were selected based on the following exclusion criteria for studies:

- Focusing exclusively on placebo stimulation;
- Being limited to experimental clinical protocol presentation;
- Not reporting EEG analysis results;
- Not including specific EEG-tES interaction analysis;
- Using exclusively animals or phantom models for tES treatment analysis;
- Lacking information on electrode localization or not reporting, at least, the anode position;
- Publications exclusively analyzing or commenting on experimental research (e.g., reviews, commentaries, or editorials).

### 2.3. Quality Assessment Strategy

The papers were evaluated using the Quality Assessment Tool for Quantitative Studies (QATQS) [16], developed by researchers from Canada’s Efficient Public Health Practice Project (EPHPP).

Specifically, the six components of the QATQS were considered: (i) selection bias, (ii) study design, (iii) confounders, (iv) blinding, (v) data collection methods, and (vi) withdrawal and dropouts. These components incorporate the criteria outlined in the Cochrane Collaboration and PRISMA declaration guidelines concerning bias issues [13,17]. Each component was rated by assigning a quality score ranging from 1 to 3. The individual component ratings were first assessed, and an overall score was then calculated for each article. Papers were classified as strong when no component received a score of 3. A single component with a score of 3 led to a moderate classification. Articles with two or more components scoring 3 were classified as weak.

The initial evaluation was conducted by the second author, adhering to the QATQS protocol guidelines. Subsequently, the third author independently reassessed the papers. In cases of disagreement, all authors participated in discussions to reach a consensus. A QATQS dictionary was used to ensure consistency and standardization of the results. After further discussions, the authors confirmed the absence of discrepancies in outcome interpretation.

## 3. Results

**Figure 2:**
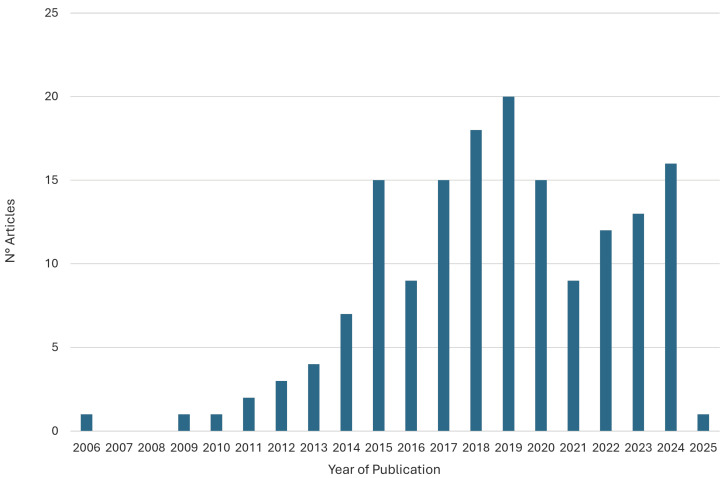
Temporal trend in publication years of the included studies.

A comprehensive literature search was conducted through Google Scholar, Scopus, PubMed, ScienceDirect, Web of Science, and IEEE Xplore, resulting in the identification of 719 records. Of these, 453 records were excluded due to duplication or non-compliance with the predefined pre-screening criteria. Subsequently, during the screening phase, an additional 87 articles were excluded due to irrelevance to the established search query. Furthermore, 85 articles were removed during the eligibility assessment, as they did not meet the predefined inclusion criteria. Manual screening of reference lists from studies meeting the eligibility criteria ensured more comprehensive coverage of the literature. This approach yielded 60 additional relevant records not captured by the initial database search. Moreover, since the described procedure initially yielded only two contributions relevant to RQ-II, a modified search was conducted to identify additional references [18]. The word *loop* was added to the previous query, and the search was extended to the abstract and keyword fields. Finally, other types of contributions, such as letters, were also considered. This modified search strategy yielded eight additional references for RQ-II. Overall, 162 studies were ultimately included in the review analysis. The distribution of the selected articles by year of publication is illustrated in Figure 2.

The included articles were systematically categorized and analyzed according to the specific research questions they addressed. For each category, a detailed table was constructed, containing information on the clinical use case, waveform type, anode and cathode positioning, the number of tES electrodes employed, the QATQS index, sample size, the analyzed EEG features, and the applied data analysis methods.

The clinical populations examined in the reviewed studies demonstrate significant heterogeneity, encompassing a wide range of neurological disorders, including epilepsy, Alzheimer’s disease, Parkinson’s disease, and stroke, as well as psychiatric and neurodevelopmental disorders. The majority of studies focus on tDCS, with a smaller subset examining tACS and a clear minority exploring alternative waveform modalities such as transcranial Random Noise Stimulation (tRNS) or transcranial Pulsed Current Stimulation (tPCS). Electrode placement—whether anodal or cathodal—varies significantly across studies, frequently targeting the left or right Dorsolateral Prefrontal Cortex (DLPFC) or the Epileptogenic Focus (EF) in research involving epileptic patients. Quality assessment reveals a predominance of studies rated as “weak”, while only a limited number receive “moderate” or “strong” ratings, often associated with relatively small sample sizes. From an analytical perspective, most studies use traditional statistical methods, with only a few employing machine learning or deep learning approaches for EEG analysis.

### 3.1. Results for Research Question I (RQ-I)

Table 1 presents the articles addressing the first research question—namely, whether EEG is used to guide tES treatment. The investigated clinical conditions include epilepsy [19,20], Alzheimer’s disease [21], and chronic tinnitus [22], in addition to a study involving a cohort of healthy subjects [23]. The majority of the studies focus on the use of tDCS, with only one employing tACS. The QATQS evaluation indicates one study as “strong”, with the remaining studies categorized as “moderate” or “weak”. Conventional statistical approaches are predominantly used, with machine learning techniques applied in only one instance for EEG data analysis.

The table illustrates how EEG primarily helps in setting either the electrode placement or the stimulation frequency. For example, parameters such as functional connectivity and the localization of the epileptic focus are used to determine the optimal stimulation site, while the Individual Alpha Frequency (IAF) is employed to identify the ideal stimulation frequency for tACS. The IAF refers to the most prominent (in terms of power) frequency of alpha oscillations. To ensure consistency across this study, the term IAF is used throughout, even when the original studies refer to peak alpha frequency, as the two are considered synonymous. This approach proposes a methodological shift, wherein the stimulation protocol is customized to the individual’s specific neurophysiological characteristics rather than relying on standardized placements or general models, thereby enabling more precise and potentially more effective stimulation.

### 3.2. Results for Research Question II (RQ-II)

Table 2 presents the 10 studies addressing RQ-II—namely, whether EEG is used to guide tES in real time. For each study, the clinical target, stimulation waveform, anode and cathode position, number of stimulation electrodes, QATQS score, and sample size are reported. The adaptive rule proposed in each study is also summarized, specifying the EEG-based adaptation condition and the subsequently adjusted tES parameters. With respect to the clinical use case, nine studies were conducted on healthy participants and one on patients with MDD. Regarding stimulation parameters, nine studies employed tACS, whereas only one used tDCS. The most common configuration consisted of 2 stimulation electrodes (seven studies), followed by 3 electrodes (two studies), and a single case employing 32 electrodes. Methodological quality, assessed through QATQS, was rated as weak in six studies, medium in two, and strong in two. Sample sizes ranged from 10 to 60 participants. Regarding adaptive stimulation parameters, adjustments targeted the onset (five studies), frequency (four studies), phase (two studies), amplitude (one study), and stimulation site (one study), with some protocols implementing concurrent modifications of multiple parameters. Four studies [24,25,26,27] applied tACS during sleep to enhance declarative memory consolidation or improve metamemory in healthy participants. The onset, frequency, and phase of stimulation were adapted based on the online detection of EEG Slow Waves (SWs), in the frequency range of (0.1–1.2) Hz. SWs consist of slow, synchronized upward and downward deflections of the EEG, associated with declarative memory consolidation. SW activity was estimated through a virtual EEG channel obtained by averaging 13 fronto-central electrodes, providing a measure of the overall synchronous activity. The algorithm calculated the ratio between the cumulative power in the SW range and the total power in the range of (0.1–250.0) Hz. Stimulation was triggered when this ratio exceeded a predefined threshold, set to 20% [24,25] or 30% [26,27] of the total power in the range of (0.1–250) Hz. In addition, the tACS frequency was set to the peak frequency within the SW range, and the stimulation phase was aligned with the ongoing SW activity so that the current peak coincided with the up states (positive half-waves) of the endogenous oscillations, as determined based on the averaged fronto-central EEG signal. However, the four papers differ in cognitive and neurophysiological targets. In particular, Ketz et al. (2018) [24] and Jones et al. (2018) [25] aimed to enhance declarative memory consolidation by applying closed-loop tACS during sleep. Participants were trained on a target detection task involving the identification of hidden objects in complex visual scenes prior to sleep, and performance was assessed the following day using identical images from training (“repeated images”) and generalized images depicting the same scenes from different viewpoints (“generalized images”). In both studies, stimulation selectively improved performance on generalized images, with no significant effect on repeated images, suggesting that closed-loop tACS facilitates schematization and integration of new information rather than simple rote recall. EEG analyses revealed that this behavioral gain correlated with transient modulation of slow-wave power and increased slow wave–spindle coupling. In addition, Jones et al. introduced the concept of dose dependence, showing an inverted U-shaped dose–response relationship: an intermediate number of SW-locked stimulations enhanced generalization, whereas excessive stimulation induced a refractory effect, reducing slow-wave power and abolishing the benefit. In contrast, Pilly et al. (2020) [27] and Hubbard et al. (2021) [26] focused on metamemory, a metacognitive function corresponding to the ability to monitor and evaluate the quality of individual memories, which is closely associated with declarative memory. In both cases, a stimulation paradigm based on STAMPs (Spatiotemporal Amplitude-Modulated Patterns) was introduced. STAMPs are unique patterns of transcranial electrical stimulation characterized by a specific spatiotemporal distribution of currents, designed to “tag” one-shot episodic experiences acquired in virtual reality (VR) and to selectively reactivate them during sleep. Specifically, in the study of Pilly et al. (2020) [27], the application of STAMPs as brief pulses during slow-wave oscillations in sleep led to a 10–20% improvement in metamemory for the targeted episodes compared to control episodes 48 h after initial encoding. However, the effect was dose-dependent, as an excessive number of stimulations produced detrimental outcomes. The observed improvements were mediated by increased power in the slow-spindle band (8–12 Hz) over left temporal regions. In contrast, Hubbard et al. (2021) [26] investigated the underlying neurophysiological mechanisms by analyzing EEG functional connectivity using graph-theoretical approaches. In this case, the findings showed that STAMPs induced an increase in theta-band connectivity and greater network efficiency in the spindle band and that changes in beta-band path length were predictive of metamemory improvements.

Motor memory consolidation in healthy participants was investigated by Lustenberger et al. (2016) [28] by focusing the effect of closed-loop tACS on spindle activity during sleep. Motor memory refers to implicit procedural memory underlying the acquisition and stabilization of motor skills and movement sequences. In this study, EEG signals from Fz and CPz were continuously monitored, and stimulation was delivered exclusively during the online detection of sleep spindles (10–16 Hz), ensuring temporal alignment with endogenous oscillatory activity. Stimulation (1.5 s bursts of 12 Hz, 1 mA) was triggered when five consecutive peaks exceeded an individually defined threshold. The threshold was determined by calculating the mean amplitude within the 10–16 Hz band during the first continuous 10 min epoch of NREM sleep and multiplying it by a subject-specific factor selected to maximize the F score, defined as the harmonic mean of precision and recall between a standard offline spindle detection algorithm and the online detection method. Results showed that feedback-controlled tACS significantly improved speed in a motor sequence task, without affecting accuracy, vigilance, or declarative memory. Sleep macro-architecture remained unchanged, whereas EEG analyses revealed a selective increase in fast spindle activity (15–16 Hz) during the light sleep stage (N2), particularly in participants exhibiting the largest motor performance gains. Moreover, the spindle activity enhancement predicted the degree of motor speed improvement, and similar correlations between fast spindle characteristics and motor learning were found, even during sham nights.

Four studies [29,30,31,32] proposed closed-loop approaches adapting the tACS phase, frequency, or onset to ongoing alpha oscillations (8–12 Hz) for investigation of (i) working memory accuracy and (ii) visual detection performance in healthy subjects, (iii) containment of depression symptoms in MDD patients, and (iv) the impact of TACS on alpha power without a specific clinical target, respectively. Haslacher et al. (2024) [29] investigated the improvement of fidelity of neural representations supporting working memory in healthy participants. In particular, alpha oscillations (8–14) Hz from the parieto-occipital cortex were recorded via a virtual electrode, computed using a Laplacian filter centered on electrode Pz, which was surrounded electrodes PO7, PO8, P3, and P4. During stimulation, the phase of the tACS envelope (8 kHz carrier frequency, ±10 mA stimulation amplitude) was continuously adapted to the phase of the endogenous alpha oscillations in real time, maintaining a constant phase lag between the two signals. In the experimental group, working memory accuracy, defined as the percentage of correct responses in the working memory task, improved most consistently at a 330° phase lag, although the optimal phase varied across participants. At the neural level, Closed-Loop Amplitude-Modulated tACS (CLAM-tACS) modulated alpha synchrony within a fronto-parietal network, and the stimulation phase enhancing synchrony matched the phase influencing working memory accuracy. Moreover, increased synchrony was associated with reduced working memory performance, whereas decreased synchrony corresponded to performance improvements, effects not observed in controls. Additionally, Stecher et al. (2021) [30] tested whether closed-loop adaptation of stimulation frequency to ongoing parietal alpha oscillations (8–12 Hz) in healthy young adults could improve visual detection performance based on the established role of alpha rhythms in visual perception and attention. In particular, Stetcher et al. proposed a closed-loop approach where the stimulation frequency was updated every 8 s based on the IAF. In this case, the IAF was estimated from EEG segments of equal duration recorded after each stimulation block and defined as the maximum value in the power spectrum between 7.2 and 12.8 Hz. The power at this maximum had to exceed the mean power across the entire band (7.2–12.8 Hz) plus one standard error to guarantee correspondence with a true spectral peak rather than noise. In the absence of a reliable peak, a default stimulation frequency of 10 Hz was applied. However, no behavioral differences emerged between groups in target detection, and analyses revealed no phase-dependent modulation of perception. In addition, IAF varied across epochs in all groups, and the adaptive system often failed to detect a stable IAF peak. Post-stimulation analyses showed that alpha power was significantly increased in the fixed-frequency group relative to the sham, whereas no difference was observed for the closed-loop group. Notably, this alpha-power enhancement in the fixed-frequency condition was associated with greater IAF stability over time. Schwippel et al. (2024) [31] conducted the only patient-based study, testing closed-loop tACS as a potential treatment for individuals with Major Depressive Disorder (MDD). The adaptive rule targeted pathological increases in alpha oscillations (8–12 Hz) in the prefrontal cortex by triggering stimulation only when frontal alpha power (IAF ±2 Hz) exceeded an individually defined threshold established from baseline EEG with eyes open and closed. During stimulation sessions, patients watched relaxing videos while receiving 120 s trains of tACS initiated whenever the threshold was crossed. A 5-day treatment course produced marked clinical benefits, with 80% of patients achieving response and remission at 2-week follow-up, along with improved quality of life. EEG analyses revealed significant reductions in alpha power that strongly correlated with symptom improvement, supporting alpha modulation as a potential biomarker of therapeutic efficacy. Finally, Zarubin et al. (2020) [32] investigated whether closed-loop tACS phase-locked to the individual alpha frequency (IAF) could transiently modulate occipito-parietal alpha oscillations in healthy adults. EEG was recorded in real time from electrode POz, and at regular intervals, an EEG segment of 250 ms was extracted, which was considered sufficient for phase estimation under the assumption that alpha oscillations remain quasi-stationary over such short timescales. Each segment was bandpass-filtered in the alpha range to estimate IAF. tACS was then delivered at the IAF, with the stimulation peak aligned to either the negative or positive peak of the alpha-band-filtered signal. Results showed reductions in alpha power detectable only in individually derived spatial components, whereas conventional electrode signals from POz and the parieto-occipital cluster showed no significant changes. The effect was stronger with eyes closed than with eyes open, restricted to the alpha frequency range, and transient, peaking immediately after stimulation and gradually decreasing over time.

Sustained attention enhancement was the focus of Caravati et al. (2024) [33]. They investigated the effects of closed-loop tDCS on fronto-central theta/alpha EEG activity in healthy university students performing the AX Continuous Performance Task (AX-CPT). A stimulation system capable of dynamically adapting both current intensity and the stimulation site to the individual’s neural state was implemented. In a preliminary phase, entropy-based metrics were computed for each participant, including approximate entropy, sample entropy, fuzzy entropy, and their multiscale extensions. These measures, suited to capture the nonlinear dynamics of EEG signals, provide insights into cognitive states, with higher values typically reflecting concentration and lower values indicating relaxation or fatigue. The channel–metric combination showing the highest discriminative power between concentration and relaxation states, as determined by Fisher’s ratio, served to train a binary classifier, with the decision threshold optimized using Youden’s index. During the experimental session, the selected EEG metric was computed every 3 s and reassessed every minute. An average value computed over 20 consecutive 3 s epochs exceeding the threshold for at least three consecutive minutes prompted a reduction in intensity of current. When the value remained below the threshold, the PSD in the theta band was computed over a 1 min segment. An increase in theta power during frontal stimulation, interpreted as possible mental fatigue or immersion, led to a change in the stimulation site if exceeding a predefined threshold of 0.1; otherwise, the stimulation current was reduced. As a result, among the channel–metric combinations, fuzzy entropy at FC6 was identified as the most sensitive marker. Most participants showed improved attentional responses, while a subgroup of non-responders with atypical theta activity did not benefit. Moreover, individualized closed-loop stimulation enhanced AX-CPT performance, yielding higher accuracy, faster and more stable reaction times, and greater efficiency than fixed or sham stimulation and counteracted the detrimental effects of mental fatigue.

**Table 1:** Articles addressing the first research questions. Expanded acronyms are reported in the Abbreviations section.

Article	Clinical Target	Waveform	A Position	C Position	N° of Electrodes	QATQS (Sample Size)	EEG Features for tES Setup	Data Analysis Method
Fregni et al. (2006) [19]	Epilepsy (seizure frequency reduction)	tDCS	Silent area	EF	2	Moderate (19)	EF	S
De Ridder et al. (2012) [22]	cCronic tinnitus (tinnitus perception reduction)	tDCS	(a) Right DLPFC; (b) based on functional connectivity	(a) Left DLPFC, (b) based on functional connectivity	2	Weak (675)	Theta and gamma functional connectivity	S
San-Juan et al. (2017) [20]	Epilepsy (interictal epileptiform discharge reduction)	tDCS	Silent area	EF	2	Weak (28)	EF	S
Marinho Andrade et al. (2023) [21]	AD (cognitive decline containment)	tDCS	(a) F5, CP5, and F4; (b) F3, P4, and P5	Contralateral; supraorbital	4	Strong (70)	PSD in all bands	ML
Mokhtarinejad et al. (2024) [23]	Healthy (time perception precision and accuracy modulation)	tACS	Oz	Cz	2	Moderate (24)	IAF and PAP	S

**Table 2:** Articles addressing the second research question. Expanded acronyms are reported in the Abbreviations section.

Article	Clinical Target	Waveform	A Position	C Position	N° of Electrodes	QATQS (Sample Size)	Adaptivity Rule
Lustenberger et al. (2016) [28]	Healthy (motor memory consolidation)	tACS	F3, F4	Cz	3	Weak (17)	Stimulation triggered when ≥5 consecutive peaks of signal filtered in (11–16) Hz > mean amplitude of baseline signal filtered in (11–16) Hz × a subject-specific constant
Ketz et al. (2018) [24]	Healthy (declarative memory consolidation)	tACS	F10	Upper contralateral arm	2	Strong (21)	Stimulation is triggered when RP (0.5–1.2 Hz) > 20% of TP. tACS frequency = peak frequency in (0.5–1.2) Hz. tACS peak aligned with the peak of the fronto-central averaged EEG signal
Jones et al. (2018) [25]	Healthy (declarative memory consolidation)	tACS	F3/F4	F3/F4	2	Weak (21)	Stimulation is triggered when RP (0.5–1.2 Hz) > 20% of TP. tACS frequency = peak frequency in (0.5–1.2) Hz. tACS peak aligned with the peak of the fronto-central averaged EEG signal
Zarubin et al. (2020) [32]	Healthy (without clinical objective)	tACS	Oz	Cz	2	Medium (20)	tACS frequency = IAF at POz. tACS peak aligned with the negative or positive peak of alpha-band-filtered signal
Pilly et al. (2020) [27]	Healthy (metamemory sensitivity improvement)	tACS	O10, TP8, P6, PO8, FT8, F6, C6, FC4, CP4, C2, P2, AF8, F2, FPz, FCz, AFz, F1, AF7, Iz, POz, P1, CPz, C1, CP3, FC3, C5, F5, FT7, PO7, P5, TP7, O9	O10, TP8, P6, PO8, FT8, F6, C6, FC4, CP4, C2, P2, AF8, F2, FPz, FCz, AFz, F1, AF7, Iz, POz, P1, CPz, C1, CP3, FC3, C5, F5, FT7, PO7, P5, TP7, O9	32	Weak (30)	Stimulation is triggered when RP (0.5–1.2 Hz) > 30% of TP. tACS frequency = peak frequency in (0.5–1.2) Hz. tACS peak aligned with the peak of the fronto-central averaged EEG signal
Stecher et al. (2021) [30]	Healthy (visual detection performance improvement)	tACS	Parieto-occipital cortex (Cz/Oz)	Parieto-occipital cortex (Cz/Oz)	2	Medium (60)	tACS frequency = IAF at Pz
Hubbard et al. (2021) [26]	Healthy (metamemory sensitivity improvement)	tACS	Frontal cortex	Frontal cortex	2	Strong (18)	Stimulation is triggered when RP (0.5–1.2 Hz) > 30% of TP. tACS frequency = peak frequency in (0.5–1.2) Hz. tACS peak aligned with the peak of the fronto-central averaged EEG signal
Caravati et al. (2024) [33]	Healthy (sustained attention enhancement)	tDCS	(a) F3; (b) P3	(a) Fp2; (b) P4	2	Weak (10)	Stimulation amplitude or stimulation site (frontal vs. parietal) is modulated depending on whether appropriate thresholds are reached by two features: a subject-specific metric and PSD in the theta band
Haslacher et al. (2024) [29]	Healthy (working memory enhancement)	tACS	Oz	Cz	2	Weak (50)	tACS peaks are phase-locked to alpha-band-filtered signal with a maintained constant phase lag
Schwippel et al. (2024) [31]	MDD (depressive symptom reduction)	tACS	F3, F4	Cz	3	Weak (10)	Stimulation triggered when 10 s IAF ±2 Hz power > 60 s baseline threshold.

### 3.3. Results for Research Question III (RQ-III)

Table 3 presents the 132 studies addressing the third research question—namely, whether the assessment of the treatment outcomes is based on EEG analysis. The table reveals a predominant use of spectral power, particularly in alpha (seventeen articles), theta (fourteen articles), and beta bands (twelve articles), as a neurophysiological feature for assessment of the effects of transcranial stimulation on brain activity. Event-Related Potentials (ERPs) were reported in eleven studies, indicating a frequent use of measures time-locked to cognitive or sensory stimuli. Furthermore, the Event-Related Desynchronization/Synchronization (ERD/ERS) parameters, observed in six and five studies respectively, reflect a less common focus on the dynamic analysis of induced brain activity. Other features, such as the Global/Local Mean Field Power (GMFP/LMFP), connectivity indices (e.g., Phase Locking Value (PLV) and Lagged Phase Synchronization (LPS)), and Approximate Entropy (ApEn), were reported in isolated cases.

The table includes 86% of the reviewed studies, highlighting the predominant use of EEG to assess the effects of tES treatments based on standardized setups. In many studies (25% of the papers in Table 3), the use of post-treatment EEG is limited to investigating the relationship between the stimulation setup and electrophysiological features. In 25% of the cases, a clinical outcome is evaluated in addition to EEG features; however, the relationship with the stimulation protocol is assessed independently. The majority of studies (35%) perform statistical analyses on the relationship between EEG outcomes and clinical outcomes, primarily aiming to identify EEG features as potential markers of clinical improvement. Only a small proportion of studies (15%) explore a neurofunctional relationship between EEG features and clinical outcomes, and even in these cases, only a limited number of clinical outcome scales are employed.

### 3.4. Results Relevant to Both Research Questions I (RQ-I) and III (RQ-III)

Table 4 presents the studies simultaneously meeting the criteria set by the first and third research questions—namely, whether EEG is used both to design stimulation parameters and to assess tES effects. Regarding RQ-I, three main EEG feature extraction approaches are generally employed in the design of tES treatments focused on (i) spectral parameters, such as abnormal patterns of absolute or relative power in theta and alpha bands; (ii) epileptogenic foci in epilepsy-related studies; or (iii) cortical sources localized by exploiting techniques like Low-Resolution Brain Electromagnetic Tomography (LORETA). The spectral domain is particularly relevant in the context of tACS, where the stimulation frequency is typically determined based on spectral EEG features. In particular, the IAF, the Individual Theta Frequency (ITF), and the EEG band exhibiting the highest relative power are the most used spectral features.

**Table 3:** Articles addressing the third research question. Expanded acronyms are reported in the Abbreviations section. n.a.: not available.

Article	Clinical Target	Waveform	A Position	C Position	N° of Electrodes	QATQS (Sample Size)	tES-Modulated EEG Features	Data Analysis Method
Palm et al. (2009) [34]	MDD (depressive symptom reduction)	tDCS	Left DLPFC (F3)	Right supraorbital area	2	Weak (1)	Absolute and relative power in delta, theta, and alpha bands	S
Zaehle et al. (2011) [35]	Healthy (working memory performance improvement)	tDCS	Left DLPFC (F3)	Ipsilateral left mastoid	2	Weak (16)	ERP and ERSP	S
Zaehle et al. (2011) [36]	Healthy (auditory discrimination improvement)	tDCS	(a) T7 or Cp5; (b) contralateral supraorbital	(a) contralateral supraorbital; (b) T7 or Cp5	2	Weak (14)	ERP	S
Kasashima et al. (2012) [37]	Stroke (motor function recovery)	tDCS	M1 of affected hemisphere	Opposite supraorbital region	2	Weak (6)	ERD	S
Kongthong et al. (2013) [38]	Healthy (semantic processing efficiency improvement, faces)	tDCS	right temporal area (T6)	Left DLPFC (F3)	2	Weak (14)	LPC and ERP	S
Rütsche et al. (2013) [39]	Healthy (arithmetic performance enhancement)	tDCS	left PPC	n.a.	2	Weak (26)	ERS/ERD	S
Lazarev et al. (2013) [40]	Healthy (without clinical objective)	HD-tDCS	C3	5cm to C3	5	Weak (15)	Amplitude spectra	S
Mangia et al. (2014) [41]	Healthy (visuospatial attention enhancement)	tDCS	Right PPC	Ipsilateral deltoid muscle	2	Weak (10)	PSD in theta, alpha, beta, and gamma bands	S
Romero Lauro et al. (2014) [42]	Healthy (visuospatial attention enhancement)	tDCS	Right PPC	Left supraorbital area	2	Weak (14)	GMFP and LMFP	S
Roy et al. (2014) [43]	Healthy (attention and processing efficiency improvement)	HD-tDCS	Between the C3 and CP3	Left sensorimotor cortex	5	Weak (8)	ERS; ERD	S
Crivelli et al. (2014) [44]	Healthy (executive functions improvement)	tDCS	Right DLPFC	Cephalic area	2	Weak (22)	ERP	n.a.
von Mengden (2014) [45]	Healthy (working memory enhancement)	tACS	F3 and F4	Mastoid	2	Weak (1)	PSD in theta, alpha, and beta bands	S
Powell et al. (2014) [46]	Affective disorder (mood symptom reduction)	tDCS	left DLPFC (F3)	F8	2	Weak (18)	Relative power in alpha and theta bands; ERP	S
Dominguez et al. (2014) [47]	Stroke (language production improvement)	tDCS	Left frontal area	Right contralateral area	2	Weak (1)	Absolute power and coherence in delta, theta, alpha, and beta bands	S
Miller et al. (2015) [48]	Healthy (working memory accuracy enhancement)	tDCS	AFz	Under the chin	2	Weak (8)	frontal–midline theta amplitude	S
D’Atri et al. (2015) [49]	Healthy (episodic memory facilitation)	osc-tDCS	(a) Fz; (b) right deltoid muscle	(a) right deltoid muscle; (b) Fz	2	Weak (20)	EEG oscillatory components	S
Jindal et al. (2015) [50]	Stroke (motor function recovery)	tDCS	Left DLPFC (F3)	Cz	2	Weak (5)	MEP and log-transformed mean power	S
Sood et al. (2015) [51]	Stroke (ischemia- related impairment containment)	tDCS	DLPFC (F3 and F4)	Cz	3	Weak (5)	Log-transformed mean power in the range of (0.5–11.25 Hz)	S
Amatachaya et al. (2015) [52]	ASD (Attention regulation support)	tDCS	Left DLPFC (F3)	Right shoulder	2	Weak (24)	Peak alpha frequency	S
Cosmo et al. (2015b) [53]	ADHD (inhibitory control and attention improvement)	tDCS	left DLPFC (F3)	right DLPFC (F4)	2	Strong (60)	Functional cortical network	S
Del Felice et al. (2015) [54]	Epilepsy (seizure burden containment; declarative memory consolidation enhancement)	so-tDCS	Frontal–temporal (F7-T3 or F8-T8)	Ipsilateral mastoid	2	Weak (12)	Spindle frequency and cortical sources	S
Hoy et al. (2015) [55]	Schizophrenia (working memory and cognitive control improvement)	tDCS	Frontal cortex (F3)	Right supraorbital	2	Weak (16)	Gamma ERS and correlation	S
Dutta et al. (2015) [56]	Stroke (motor recovery)	tDCS	Motor cortex (Cz)	Left supraorbital notch	2	Moderate (4)	Log-transformed mean power in the range of (0.5–11.25 Hz)	S
Kasashima-Shindo et al. (2015) [37]	Stroke (motor recovery)	tDCS	Primary sensorimotor cortex	contralateral supraorbital area	2	Weak (18)	ERD	S
Wu et al. (2015) [57]	Stroke (language naming improvement)	tDCS	Left posterior perisylvian	Unaffected shoulder	2	Weak (12)	ApEn	S
Jindal et al. (2015) [58]	Stroke (motor function recovery)	tDCS	Motor cortex (Cz)	Frontal cortex (F3 or F4)	2	Weak (29)	Log-transformed mean power in the range of 0.5–11.25 Hz; Relative power in all bands	S
Ang et al. (2015) [59]	Stroke (motor imagery BCI performance and motor recovery)	tDCS	M1 of the ipsilesional hemisphere	Contralesional M1	2	Moderate (19)	ERD	n.a.
Ulam et al. (2015) [60]	TBI (attention/ working memory improvement)	tDCS	Left DLPFC (F3)	Right supraorbital area (Fp2)	2	Strong (26)	Relative power in delta, theta, alpha, and beta bands	S
Impey et al. (2015) [61]	Healthy (auditory discrimination improvement)	tDCS	Left auditory cortex (between C5 and T7)	Contralateral forehead	2	Strong (12)	ERP (MMN)	S
Sood et al. (2016) [62]	Healthy (motor performance/ processing support)	tDCS	C3	FC1, FC5, CP5, CP1	5	Weak (5)	Log-transformed mean power in the range of 0.5–11.25 Hz	S
Cappon et al. (2016) [63]	Healthy (cognitive performance modulation, attention)	tACS	Fz (electrode area centroid)	C5 (electrode area centroid)	2	Weak (18)	ERS/ERD	S
Caldiroli et al. (2016) [64]	Healthy (semantic decision efficiency improvement)	tDCS	Right supraorbital region	Left DLPFC (F3)	2	Weak (30)	ERP	S
Marceglia et al. (2016) [65]	AD (cognitive symptoms mitigation)	tDCS	Bilateral temporal–parietal area	Tight deltoid muscle	3	Weak (7)	Absolute power and coherence in all bands	S
Liu et al. (2016) [66]	Epilepsy (depressive symptom reduction and memory consolidation enhancement)	tDCS	Left DLPFC (F3)	Right supraorbital area	2	Weak (37)	Relative power in alpha and theta bands	S
Dunn et al. (2016) [67]	Schizophrenia (auditory processing and working memory improvement)	tDCS	DLPFC (Fp1 and Fp2)	Right upper arm	3	Weak (36)	ERP (P300)	S
D’Agata et al. (2016) [68]	Stroke (cognitive function improvement)	tDCS	Perilesional M1(C3 or C4)	ContralesionalM1	2	Weak (34)	ERP (P300, N200)	S
Ashikhmin et al. (2017) [69]	Healthy (autonomic regulation and cognitive vigilance modulation)	tDCS	Over T3 area	Over A2 lead	2	Weak (10)	Relative power in all bands	n.a.
Angulo-Sherman et al. (2017) [70]	Healthy (motor imagery classification/ accuracy support)	tDCS	(a) In front of C3; (b) between Cz and FC1	Inion level (3 cm to the left hemisphere)	2	Weak (5)	Absolute power in the range of 9–30 Hz	S
Angulo-Sherman et al. (2017) [71]	Healthy (motor imagery classification/ accuracy support)	HD-tDCS	(a) C3; (b) Cz	(a) FC1, FC5, CP1, and CP5; (b) FC1, CP1, FC2, and CP2	5	Weak (2)	ERS	S
Grande et al. (2017) [72]	Healthy (visual working memory enhancement, aging)	tACS	Parietal cortex (P3/P4)	Parietal cortex (P4/P3)	2	Weak (19)	ERP (N200)	S
Donaldson et al. (2017) [73]	Healthy (social cognition, face processing improvement)	tDCS	Right TPJ	right TPJ		Weak (n.a.)	ERP (N170, P300)	n.a.
Berger et al. (2017) [74]	Healthy (motor learning facilitation)	tACS	Parietal cortex (P3/P4)	Parietal cortex (P4/P3)	2	Weak (15)	Relative power in alpha band	S
Cortes et al. (2017) [75]	Healthy (fatigue resistance/ perceived exertion modulation)	tDCS	Motor cortex (Cz)	Fpz	2	Weak (4)	Total EEG power in all bands	S
Romero Lauro et al. (2017) [42]	Healthy (visuospatial attention enhancement)	tDCS	Right PPC	n.a.	n.a.	Weak (14)	GMFP and LMFP on mean TEP	S
Ladenbauer et al. (2017) [76]	MCI (sleep- dependent memory consolidation enhancement)	so-tDCS	Prefrontal cortex (F3–F4)	Ipsilateral mastoid	3	moderate (16)	Absolute power in the range of 0.5–1 Hz) andfast spindles (12–15 Hz)	S
Impey et al. (2017) [77]	Schizophrenia (working memory and auditory processing improvement)	tDCS	Left auditory or left frontal cortex	Contralateral forehead	2	Weak (12)	ERP	S
Naros and Gharabaghi (2017) [78]	Stroke (motor self-regulation training improvement)	tACS	Ipsilesional sensorimotor cortex	Contralesionalforehead	2	Weak (20)	Relative power and ERD in beta band	S
Yuan et al. (2017) [79]	Stroke (swallowing apraxia improvement)	tDCS	M1	Contralateralshoulder		Weak (9)	ApEn	S
O’Neil-Pirozzi et al. (2017) [80]	TBI (immediate memory improvement)	tDCS	Left DLPFC	Right supraorbital	2	Weak (8)	Auditory ERP (P300) and absolute power in alpha and theta bands	S
Boudewyn et al. (2018) [81]	Healthy (proactive control enhancement)	tDCS	Left DLPFC	Right supraorbital	2	Weak (20)	Absolute power in gamma band	S
Kang et al. (2018) [82]	ASD (cognitive flexibility/ complexity support)	tDCS	DLPFC	Right supraorbital	2	Weak (13)	MER	S
Mane et al. (2018) [83]	Chronic stroke (motor recovery monitoring)	tDCS	The ipsilesional M1	Contralesional M1	2	Weak (19)	PRI, delta–alpha ratio, theta–beta ratio, theta–alpha ratio, theta–beta–alpha ratio, pdBSI, and Rbsi	S
Cucik et al. (2018) [84]	Healthy (alertness/state modulation)	tDCS	Left motor cortex	Contralateral eyebrow	2	Weak (16)	MSS and SV	S
Friedrich et al. (2018) [85]	Healthy (inhibitory control modulation)	tDCS	Contralateral orbit parallel to the eyebrow	Somatosensory cortex (C3)	2	Weak (17)	ERP	S
Mondini et al. (2018) [86]	Healthy (motor performance/ ERD training support)	tDCS	(a) Left motor cortex (C3); (b) right supraorbital (Fp2)	(a) Right supraorbital (Fp2); (b) left motor cortex (C3)	2	Weak (20)	Alpha-ERD and relative power in theta and alpha bands	S
Holgado et al. (2018) [87]	Healthy (exercise performance modulation)	tDCS	DLPFC	Shoulder	2	Weak (36)	Absolute power in all bands	S
Berger et al. (2018) [88]	Healthy (motor learning facilitation)	tACS	P3	P4	2	Weak (24)	Relative power in alpha band	S
Ferrucci et al. (2018) [89]	Dementia (cognitive symptoms mitigation)	tDCS	Fronto-temporal (F7–F8)	Right deltoid muscle	3	Moderate (13)	Absolute power in alpha and beta bands	S
Shahsavar et al. (2018) [90]	Depression (depressive symptom reduction)	tDCS	Left DLPFC (F3)	Right DLPFC (F4)	2	Weak (7)	ERP and average alpha energy	S
Meiron et al. (2018) [91]	Epilepsy (seizure frequency reduction)	HD-tDCS	PO3-P6- AF3-F6- FC4-O1 CP3-C1- FC8-C6- FCz-FC3 O4-F2-CP4 PO4-O2 AF8-C2	C2, TP8,CP8, O3, T8	24	Weak (1)	Mean number of spikers, mean peak amplitude, and mean absolute power	S
Rassovsky et al. (2018) [92]	Schizophrenia (face processing and attention improvement)	tDCS	DLPFC (F3)	Right supraorbital (Fp2)	2	Weak (38)	ERP (P300 and N170)	S
Hordacre et al. (2018) [93]	Stroke (motor network reorganization support)	tDCS	M1	Contralateral orbit	2	Weak (10)	Connectivity in delta, theta, alpha, beta, and gamma bands	S
Nicolo et al. (2018) [94]	Stroke (motor function recovery)	tDCS	Ipsilesional supraorbital region	ContralesionalM1	2	moderate (41)	Effective and functional connectivity	S
Straudi et al. (2019) [95]	MCS (arousal/ awareness support)	tDCS	M1	M1	n.a.	Weak (10)	Parietal site EEG upper alpha bandwidth	S
D’Atri et al. (2019) [96]	Healthy (oscillatory cognitive performance modulation)	tACS	Right fronto-temporal area	Left fronto-temporal area	2	Moderate (20)	Relative power in all bands	S
Dondè et al. (2019) [97]	Healthy (sustained attention enhancement)	tRNS	right-DLPFC (F4)	Left DLPFC (F3)	2	Strong (19)	Beta/alpha power ratio	S
Donaldson et al. (2019) [98]	Healthy (target detection/ attention improvement)	tDCS	rTPJ	rTPJ	n.a.	Weak (n.a.)	ERP (P300)	n.a.
Dowsett et al. (2019) [99]	Healthy (visual steady-state detection performance modulation)	tACS	Cz	O2	2	Weak (30)	SSVEP	S
Bueno-Lopez et al. (2019) [100]	Healthy (sleep-related cognitive consolidation support)	so-tDCS	Prefrontal positions (F3–F4)	Ipsilateral mastoids (M1–M2)	4	moderate (23)	Relative power in all bands	S
Handiru et al. (2019) [101]	Stroke (motor recovery, bilateral coordination)	tDCS	Ipsilesional M1	contralesionalM1	n.a.	Weak (19)	Beta coherence	S
Willms et al. (2019) [102]	Healthy (attentional control modulation)	tDCS	Left DLPFC	right DLPFC	n.a.	Weak (n.a.)	Power in alpha band	S
Mastakouri et al. (2019) [103]	Healthy (motor performance/ learning support)	HD-tACS	M1 (C3)	Cz, F3, T7, and P3	5	Moderate (19)	Absolute power in beta band	S
Emonson et al. (2019) [104]	MCI (cognitive function/TEP monitoring)	tDCS	DLPFC (F3)	Contralateral supraorbital (Fp2)	2	Weak (49)	ERP and TEP	S
Cespòn et al. (2019) [105]	AD (cognitive symptoms mitigation)	tDCS	left DLPFC (F3)	Right shoulder	2	moderate (26)	ERP and absolute power in theta, alpha, and beta bands	S
Alexander et al. (2019) [106]	MDD (depressive symptom reduction)	tACS	left/right DLPFC (F3/F4)	Cz	2	Strong (32)	Absolute power in alpha band	S
Meiron et al. (2019) [107]	Epilepsy (seizure frequency containment)	HD-tDCS	frontal–parietal cortex (AF8, F2, C2, PO4)	C6	5	Weak (1)	Relative power in theta, alpha, and beta bands; delta-ERD	S
Ahn et al. (2019) [108]	Schizophrenia (cognitive control and network modulation)	tACS and tDCS	Prefrontal cortex (between F3 and Fp1)	TPJ (between T3 and P3)	2	moderate (22)	Alpha oscillations, PSD, and functional connectivity	S
Singh et al. (2019) [109]	Schizophrenia (negative symptoms/ cognitive slowing mitigation)	tPCS	Cerebellar vermis	Right shoulder	2	Weak (9)	Relative power in delta and theta bands	S
Schoellmann et al. (2019) [110]	PD (motor symptom relief)	tDCS	left sensorimotor (C3)	Right frontal area (FP2)	2	Moderate (21)	Relative power and coherence in all bands	S
Mane et al. (2019) [111]	Stroke (motor recovery tracking)	tDCS	ipsilesional M1	ContralesionalM1	2	Weak (19)	PSD and relative power in delta, theta, alpha, and beta bands; PRI; rBSI	S
Bao et al. (2019) [112]	Stroke (motor function recovery)	HD-tDCS	Ipsilesional M1 (C3)	Frontal–parietal cortex (F1, F5, P1, P5)	5	Weak (30)	coherence and PSD in alpha, beta, and gamma bands	S
Luna et al. (2020) [113]	Healthy (attention/ executive modulation, PPC/DLPFC)	HD-tDCS	(a) Right PPC; (b) right DLPFC	(a) Right PPC; (b) right DLPFC	5	Moderate (92)	Absolute and relative power in alpha band	S
El-Hagrassy (2020) [114]	Healthy (executive function and attention modulation)	tDCS	Left DLPFC	(a) Right shoulder; (b) right DLPFC	2	Weak (24)	PSD in delta, theta, alpha, beta, and gamma bands	S
de Melo et al. (2020) [115]	Fibromyalgia (pain symptom reduction)	tDCS	Left M1 (C3)	Right supraorbital	2	Strong (31)	Absolute power in the range of 0.5–30 Hz	S
Sergiou et al. (2020) [116]	Substance dependence (craving regulation support)	HD-tDCS	Fpz	AF3, AF4, F3, Fz and F4	6	Weak (50)	LPP	S
Pross et al. (2020) [117]	Schizophrenia (alpha-linked cognitive improvement, exploratory)	tDCS	DLPFC	DLPFC	n.a.	Weak (40)	Alpha activity	n.a.
Gangemi et al. (2020) [118]	AD (cognitive symptoms mitigation)	tDCS	Left fronto-temporal lobe (F7-T3)	Right frontal lobe (Fp2)	2	Moderate (26)	Alpha/beta/theta rhythm	S
Nikolin et al. (2020) [119]	Depression (depressive symptom reduction; memory/ attention support)	tDCS	Left DLPFC (F3)	Right shoulder	2	Weak (20)	PSD in alpha and theta bands; ERP	S
Breitling et al. (2020) [120]	ADHD (response inhibition and attention improvement)	tDCS/ HD-tDCS	Right inferior frontal gyrus (F8)	Contralateralsupra-orbital	2 (5 for HD)	Weak (15)	ERP (N-200 and P-300)	S
Boudewyn et al. (2020) [121]	Schizophrenia (proactive control/attention enhancement)	tDCS	Left DLPFC (F3)	Right supraorbital (Fp2)	2	Moderate (37)	Relative power in gamma band	S
Jahshan et al. (2020) [122]	Schizophrenia (visual processing efficiency improvement)	tDCS	Central occipital cortex	Right shoulder	n.a.	Weak (27)	VEP	n.a.
Zhang et al. (2020) [123]	TBI (cognitive function support: attention/ memory)	tDCS	Left DLPFC (F3)	neck/F4	2	Weak (10)	ApEn; C-ApEn	S
Grasso et al. (2021) [124]	Healthy (attention/ executive function enhancement)	tDCS	Left PPC	Upper part of the right arm	2	Moderate (32)	ERP and TEP	S
Hasballah (2021) [125]	Post stroke (executive function and attention support)	tDCS	Left DLPFC (F3)	Right DLPFC (F4)	2	Weak (23)	Absolute and relative power, delta–theta–alpha–beta and delta–alpha ratios	S
Ghin et al. (2021) [126]	Healthy (visual processing enhancement, VEP)	hf-tRNS	PO3/P04	PO4/PO3	2	Weak (16)	PSD; VEP	S
Mostafavi et al. (2021) [127]	OUD (craving and relapse risk reduction support)	tDCS	(a) Left DLPFC (F3); (b) right DLPFC (F4)	(a) Right DLPFC (F4); (b) left DLPFC (F3)	2	Moderate (30)	Absolute power, amplitude, and coherence in all bands	S
Mai et al. (2021) [128]	Healthy (auditory encoding fidelity enhancement)	tDCS	Left/right auditory cortex (T7/T8)	Contralateral forehead	2	Strong (90)	EFR	S
Wang et al. (2021) [129]	Stroke (motor function recovery)	tDCS	(a)/(c) Ipsilesional M1 (C3 or C4); (b) lateral supraorbital	(a) Lateral supraorbita; (b)/(c) contralateralM1	2	Weak (19)	PSD and relative power in delta, theta, alpha, and beta bands	S
Hu et al. (2021) [130]	Healthy (attention/ executive modulation)	tACS	DLPFC (F3/F4)	DLPFC (F4/F3)	2	Weak (44)	Absolute power in alpha band; ERP	S
Ghafoor et al. (2022) [131]	Healthy (attention/ executive modulation)	HD-tACS/HD-tDCS	FpZ	Left and right PFC	5	Weak (15)	Relative power in alpha and beta bands	S
Wang et al. (2022) [132]	Ischemic stroke (motor function recovery)	tDCS	(a)/(c) Ipsilesional M1; (b) lateral supraorbital	(a) Lateral supraorbital; (b)/(c) contralateralM1	2	Moderate (32)	PSD and relative power in delta, theta, alpha, and beta bands	S
Liu et al. (2022) [133]	UWS (arousal/ awareness facilitation)	tDCS	(a) Prefrontal area; (b) left FTPC; (c) right FTPC; (d) left DLPFC	(a) Neck; (b)/(c) back of the opposite shoulder; (d) F4	2	Strong (85)	c-ApEn	S
Kim et al. (2022) [134]	PTSD (PTSD symptom reduction: intrusive/ arousal)	tDCS	Left DLPFC (F3)	Right DLPFC (F4)	2	Weak (48)	PSD in delta, theta, alpha, and beta bands	S+ML
Westwood et al. (2022) [135]	ADHD (attention and impulsivity improvement)	tDCS	F8	Right supra-orbital (Fp1)	2	Moderate (29)	PSD in alpha, theta, and beta bands	S
Maimon et al. (2022) [136]	DOC (consciousness detection/ support)	tDCS	Left DLPFC (F3)	Right supra-orbital (Fp2)	2	Weak (6)	MMN, ERP, and VC9 activity; Relative theta power	S+ML
Ayub et al. (2022) [137]	Healthy (motor task performance improvement)	tDCS	Cz	Cp1	2	Weak (10)	ERDs	S
Palmisano et al. (2022) [138]	AD (cognitive symptoms mitigation)	tACS	6 locations covering 4 lobes in both hemispheres	6 locations covering 4 lobes in both hemispheres	n.a.	Weak (15)	Spectral power in all bands; theta, alpha, and beta activity	S
Cheng et al. (2022) [139]	OCD (compulsive symptom reduction)	tDCS	AF8, AF4, AFZ, and FPZ	Right supraorbital (Fp2)	5	Weak (51)	TEP ( N45, P60, N100, and P200)	S
Wang et al. (2022) [132]	Stroke (executive/ attention support post stroke)	tDCS	Left DLPFC (F3)	Right DLPFC (Fp2)	2	Moderate (4)	Relative power in delta, theta, alpha, and beta bands	S
de Souza Moura et al. (2022) [140]	Head and neck cancer (fatigue/ cognitive symptom mitigation)	tDCS	F4	C5	2	Weak (2)	PLI; PSD at 4/8/16/24 Hz	S
Mosayebi-Samani et al. (2023) [141]	Healthy (motor cortex excitability/skill learning support)	tDCS	(a) C3; (b) F3	Contralateral supraorbital	2	Moderate (18)	TEP; TMS-evoked oscillations; MEP	S
Yeh et al. (2023) [142]	Schizophrenia (cognitive control network modulation)	tACS	(a) F1, F5, AF3, and FC3; (b) P1, P5, CP3, and PO3	(a) CPz; (b) FCz	10	Strong (35)	LPS and connectivity	S
Dagnino et al. (2023) [143]	Healthy (sustained attention/ executive enhancement)	tDCS	(a) Left DLPFC (F3, AF3, and AF7); (b) frontal gyrus (FC6 and F8)	(a) Fp2 and T7; (b) Fp2, T8, and C6	5	Strong (56)	Relative power in all bands	S+ML
Sergiou et al. (2023) [144]	Substance dependence (craving regulation support)	HD-tDCS	Fpz	vmPF (AF3, AF4, F3, Fz and F4)	6	Moderate (50)	Beta activity; Alpha and beta synchronicity	S
Kim et al. (2023) [145]	PTSD (PTSD symptom reduction)	tDCS	F3	F4	2	Weak (48)	EEG spectrogram	DL
Roy et al. (2023) [146]	Healthy (cognitive control/ attention enhancement)	tDCS	DLPFC	DLPFC	n.a.	Weak (72)	ERP	S
Liu et al. (2023) [147]	Stroke (motor function recovery)	tDCS	Ipsilesional M1	Ipsilesional M1	2	Weak (15)	PSD in all bands	S
Fabio et al. (2023) [148]	PD (executive function and motor symptom relief)	tDCS	Left DLPFC (F7)	Right supraorbital area	2	Weak (30)	PDS and absolute power in alpha and beta bands; ERP (P300 latency)	S
Chan et al. (2023) [149]	ASD (social cognition/ attention support)	tDCS	Right DLPFC (Fp2)	Left DLPFC (F3)	2	Moderate (60)	Theta E/I balance	S
Murphy et al. (2023) [150]	MDD (depressive symptom reduction)	tDCS/tRNS	Left DLPFC (F3)	Right supraorbital	2	Moderate (49)	ERS/ERD	S
Wang et al. (2023) [151]	Healthy (executive control/ attention trajectory modulation)	tACS	left DLPFC (F3)	Right DLPFC (F4)	2	Moderate (40)	Brain activity trajectories	S
Wang et al. (2024) [152]	DoC (consciousness restoration support)	HD-tDCS	Pz	Parietal cortex	5	Weak (8)	PSD and relative power in all bands; spectral, spectral exponent	S
Tarantino et al. (2024) [153]	DoC (consciousness restoration support)	tDCS	Left DLPFC	Right supraorbital	2	Weak (19)	Alpha/theta power ratio	S
Vimolratana et al. (2024) [154]	Stroke (motor function recovery)	tDCS	Lesioned hemisphere (C3/C4)	Contralateralsupraorbital	2	Moderate (34)	Absolute power in delta, theta, alpha, and beta bands	S
Singh et al. (2024) [155]	MDD (depressive symptom reduction)	tDCS	Left DLPFC (F3)	Left FTPC and FCPC	5		PSD in all bands; functional connectivity	S
Couto et al. (2024) [156]	Comorbid anxiety– depression (anxiety and depressive symptom reduction)	tDCS	(a) rVLPFC (F6); (b) vmPFC and anterior cingulate cortex (AF3)	(a) contralateral (Fp1); (b) contralateral mastoid (TP1)	2	Weak (20)	Absolute power in all bands; functional connectivity; alpha activity	S
Liu et al. (2024) [157]	Stroke (motor function recovery)	tDCS	Ipsilesional M1 (C3/C4)	Contralesional site (FP1/FP2)	2	moderate (36)	Absolute power in alpha	S
Wynn et al. (2024) [158]	Healthy (working memory and attention enhancement)	tACS	AF4 and P5	Cz	3	Weak (54)	Absolute power and peak frequency in theta and gamma bands	S
Yeh et al. (2024) [159]	Schizophrenia (default-mode connectivity normalization; cognitive symptom support)	tDCS	Left DLPFC (F3)	Fp1, Fz, C3, and F7	5	Moderate (59)	delta DMN connectivity and LPS	S
Zhou et al. (2024) [160]	Healthy (motor performance improvement)	tDCS	motor cortex	n.a.	2	Weak (29)	Relative power in alpha and beta band	S
Zhang et al. (2024) [161]	Healthy (visual steady-state detection performance)	tDCS	Oz	Cz	2	Weak (13)	SSVEP	S
Xiao et al. (2025) [162]	Bipolar depression (depressive symptom reduction)	tDCS	Left DLPFC (F3)	Right DLPFC (F4)	2	Weak (20)	Absolute power in all bands; PLV	DL

Among the articles presented in Table 4, the studies by Del Felice et al. [54] and Rocha et al. [163] reported the highest QATQS scores. The former used EEG within an intra-subject framework to personalize tACS parameters on each PD patient. For each patient, the relative power in the delta, theta, alpha, and beta bands is compared to thresholds derived from data acquired in a control group, allowing for the identification of cortical regions exhibiting significant deviations. The frequency and localization of tACS are then determined based on the extent of deviation from the normative condition. Specifically, 4 Hz-tACS is applied when fast frequencies predominate, whereas 30 Hz-tACS is used in the presence of higher relative power at slow frequencies. Regarding localization, electrodes are positioned over the scalp region showing the greatest deviation from normative values in the predominant frequency band, with the return electrode placed on the ipsilateral mastoid. Post-treatment EEG acquisition is performed at two different time points—namely, right after (T1) and 4 weeks after (T2) tACS treatment. TES effectiveness is assessed by comparing the relative powers of six regions of interest (three for each hemisphere) with respect to the pre-treatment values. Patients exhibiting excessive beta power showed a significant reduction in beta activity following 4 Hz tACS over the sensorimotor and left parietal areas at T1 and over the right sensorimotor and left frontal areas at T2. In contrast, 30 Hz tACS produced no significant effects. These results suggest effective modulation of pathological high-frequency activity in Parkinson’s disease patients through low-frequency tACS. However, there is no evidence supporting the efficacy of high-frequency tACS in patients with predominant low-frequency abnormalities.

Similarly, Rocha et al. [163] employed EEG to identify the optimal cortical target for tDCS aimed at enhancing shooting performance. EEG recordings acquired during a target shooting task performed by skilled shooters showed the highest cortical activation over the right DLPFC. For this reason, this region was then selected for anodal tDCS in unskilled participants. After tDCS, EEG showed increased beta PSD in the left DLPFC and bilateral parietal cortices and increased low-gamma PSD in the right DLPFC, interpreted as markers of improved visuospatial attention and working memory. Behavioral data confirmed improvements in both accuracy and shot grouping, linking neurophysiological and behavioral changes. The other articles offer valuable insights into the adaptation of tES parameters and their subsequent evaluation using electroencephalographic data, despite receiving low scores according to the QATQS indices. For instance, the study by Akturk et al. (2022) [164] does not account for potential confounding variables, nor does it clearly report the level of blinding applied in the experimental protocol. Nevertheless, it is notable for including the largest sample size among the studies included in the table and for proposing an interesting adaptive stimulation setup. In particular, the stimulation frequency is set at ITF −1 Hz based on the hypothesis of improved memory capacity in healthy participants through *theta–gamma coupling*, obtained by slowing the theta frequency and allowing for the integration of multiple gamma cycles within each theta cycle. The result was an increase in resting-state theta coherence around the stimulation site (F3–P3), which was associated with improved memory performance.

In epilepsy-related studies, only San-Juan et al. (2016) [20] applied tACS treatment, with a stimulation frequency of 3 Hz to match the patient’s spike–slow-wave activity and targeting the stimulation site based on the most active epileptiform zone identified through visual EEG inspection. In this case, the intervention led to clinical worsening, with a 75% increase in seizure frequency. In contrast, the remaining studies employed cathodal tDCS and consistently reported clinical improvement. These studies used EEG to identify the EF, serving as the basis for selecting the optimal stimulation site for each patient. Overall, cathodal tDCS was associated with a significant reduction in the frequency or amplitude of Epileptiform Discharges (EDs) in the stimulated cortical area [165,166,167,168].

A singular case is presented by Dallmer-Zerbe et al. [169], involving ADHD subjects with reduced amplitude of P300 in Pz. In this context, the tACS current was set with respect to both frequency and stimulation timing to promote an increase in P300 amplitude measured during a visual oddball task. Specifically, the stimulation frequency was individually tailored to match each participant’s P300 oscillatory frequency, averaging approximately 3 Hz across the subjects. Furthermore, the stimulation timing was synchronized to keep the current in phase with the P300 latency. The results show a significant increase in P300 amplitude and a reduction in errors during the cognitive task. In conclusion, the studies presented in the table reflect an active phase of research on non-real-time, closed-loop protocols, highlighting the potential of systems based on direct interaction between EEG and tES. These systems aim to enhance both cognitive performance in healthy individuals and clinical outcomes in patients, developing highly personalized treatment strategies.

### 3.5. Results for Research Question IV (RQ-IV)

The most recurrent stimulation paradigm reported in the literature was identified to address RQ-IV, with the aim of enhancing the statistical power of cross-study comparison. In this review, a homogeneous stimulation cluster was defined when three parameters coincided: current waveform, anode placement, and cathode placement.

Among the 162 articles included in this review, the most commonly used stimulation protocol involved a direct current waveform, with the anode positioned over F3 and the cathode over Fp2 according to the 10/20 International EEG system. Thirteen studies adopted this configuration, but the assessed EEG features varied considerably, including, for example, absolute power and Event-Related Synchronization (ERS%), among others. Moreover, for the same EEG feature, different adjacent channels were considered. For this reason, the analysis focused on regional effects rather than specific EEG channels to enable meaningful comparisons across studies.

The comparative analysis summarized in Table 5 highlights the absolute power in the delta, theta, alpha, and gamma frequency bands as the most commonly assessment parameter adopted to evaluate the effects of tES across different populations. Specifically, absolute gamma power in the frontal area has frequently been assessed, typically showing increased activity following stimulation. For example, Boudewyn et al. (2018) [81] report increases in electrodes FC1, Fz, and FC2; Andrade et al. (2023) [21] identify changes in Fc1 and F8 among responders; and Boudewyn et al. (2020) [121] observe widespread frontal gamma power enhancement, particularly in F3, F7, and FC5. Although all studies consistently focused on the frontal area, the different spatial resolutions of EEG limit the precision in localizing neural sources, leading to variability in the specifically identified electrode sites. This methodological constraint must be considered when interpreting the apparent consistency across findings, as the regions showing increased gamma power do not fully overlap in terms of electrode selection. In contrast, the P300 component has been explored in only a few studies, including those by O’Neil-Pirozzi et al. (2017) and Rassovsky et al. (2018) [80,92], using electrodes placed at Cz and Fz, respectively. These studies reported divergent findings, with only one showing a statistically significant effect.

**Table 4:** Articles addressing the first and third research questions. Expanded acronyms are reported in the Abbreviations section. n.a. = not available.

Article	Clinical Target	Waveform	A Position	C Position	N° of Electrodes	QATQS (Sample Size)	EEG Features for tES Setup	tES-Modulated EEG Features	Data Analysis Method
Zaehle et al. (2010) [170]	Healthy (visual attention modulation)	tACS	PO9/PO10	PO10/PO9	2	Weak (20)	IAF	Absolute power in alpha band	S
Faria et al. (2012) [165]	Epilepsy (epileptic seizure reduction)	tDCS	CPF (FP1, FPz, and FP2)	CP6 or CP5	4	Weak (17)	EF	Average number of EDs	S
Auvichayapat et al. (2013) [166]	Epilepsy (epileptic seizure reduction)	tDCS	contralateralshoulder area	EF	2	Weak (36)	EF	Average number of EDs	S
San-Juan et al. (2016) [20]	Epilepsy (epileptic seizure reduction)	tACS	frontal cortex (Fp1/Fp2)	Frontal cortex (Fp2/Fp1)	2	Weak (1)	EF	Spike-low, poli spiker-slow, slow rhythmic waves	n.a.
Stecher et al. (2017) [171]	Healthy (visual detection performance modulation)	tACS	Cz	Oz	2	Weak (33)	IAF	Absolute alpha power	S
Khayyer et al. (2018) [172]	MDD (depressive symptom reduction)	tDCS	Left/right DLPFC (F3/F4)	Cz	2	Weak (9)	LORETA EEG source localization	Absolute alpha power	S
Lin et al. (2018) [167]	Epilepsy (epileptic seizure reduction)	tDCS	controlateral shoulder	EF	2	Weak (9)	EF	PLI in delta, theta, alpha, and beta bands	n.a.
Tecchio et al. (2018) [168]	Epilepsy (epileptic seizure reduction)	tDCS	Opposite homologous	EF	2	Weak (6)	EF	Functional connectivity	S
P.-De Koninck et al. (2019) [173]	Healthy (alpha- mediated attention enhancement)	tACS	(a) PO7/PO8; (b) F3/F4	(a) PO8/PO7; (b) F4/F3	2	Weak (12)	IAF or ITF	Absolute alpha power	S
Del Felice et al. (2019) [174]	PD (motor and cognitive performance improvement)	tACS + tRNS	Based on power spectral difference	Ipsilateral mastoid	2	Moderate (15)	Relative power difference	Delta, theta, alpha, and beta power	S
Rocha et al. (2020) [163]	Healthy (shooting performance improvement)	tDCS	Contralateral supraorbital area	Right DLPFC (F4)	2	Moderate (60)	EEG activity	PSD in beta and gamma bands	S
Dallmer-Zerbe et al. (2020) [169]	ADHD (attention and inhibition improvement)	tACS	C3, C4, CP3, CP4, P3, and P4	T7, T8, TP7, TP8, P7, and P8	12	Weak (18)	P300 and ERSP max	ERP (P-300)	S
Aktürk et al. (2022) [164]	Healthy (working memory enhancement)	tACS	F3	P3	2	Weak (46)	ITF	Theta power and theta ERP connectivity	S
Radecke et al. (2023) [175]	Healthy (spatial attention enhancement)	tACS	Parietal cortex	Parietal cortex	6	Weak (22)	Alpha power lateralization	ERP	S
Gòral-Pòlrola et al. (2024) [176]	Burnout syndrome (burnout symptom reduction)	tDCS	F7	n.a.	2	Weak (1)	Alpha rhythm	EEG spectra and ERP	S
Kim et al. (2024) [177]	Healthy (inhibitory control performance enhancement)	tACS	F5 or Fpz	F7, F3, and AF7 or Afz, Fz, and FCz	4	Weak (24)	ITF	Absolute theta power	S

**Table 5:** Articles including the same type of stimulation (tDCS), with the anode placed on F3 and cathode placed on Fp2, are reported. Expanded acronyms are reported in the Abbreviations section.

Author	Sample Type	EEG Features	Results
Boudewyn et al. (2018) [81]	20 healthy, 17 female, mean age 21, range (18–30 y.o.)	Absolute power in low-gamma and high-gamma frequency bands in frontal (FC1, Fz, and FC2), central (CP1, Cz, and CP2), and posterior (PO3, Pz, and PO4) regions	Increased frontal gamma power for B cues
Andrade et al. (2023) [21]	70 AD, sex and age not reported	Absolute power of the delta, theta, alpha, beta, and gamma frequency bands in Fc1, Fc2, Fc5, Fc6, Fp1, Fp2, F3, F4, F7, F8, FT9, FT10, C3, C4, CP1, CP2, CP5, CP6, T7, T8, P3, P4, P7, P8, O1, and O2	Increased absolute power in Fc1, F8, CP5, Oz, andF7 in responder patients
Boudewyn et al. (2020) [121]	37 schizophrenia patients, 12 female, mean age 22.76 ± 3.65, range (18–30 y.o.)	Absolute power in gamma band in left frontal (F3, F7, FC5), mid frontal (AF4, AF3, Fz), right frontal (F4, F8, FC6), central (FC2, Cz, CP2, FC1, CP1), left posterior (P3, CP5, P7), mid posterior (O1, Oz, O2), and right posterior (P4, CP6, P8) regions	Increased absolute gamma power compared to the sham condition in all clusters, except the left posterior and mid posterior, when sham performed before active stimulation
Liu et al. (2016) [66]	37 epilepsy patients, sex not reported, range (18–70 y.o.)	Averaged absolute power values in delta, theta, low alpha, high alpha, beta, and low gamma bands across fronto-central (Fp1, Fp2, F3, F4, C3, and C4), left temporal (F7, T3, T5, and A1), right temporal (F8, T4, T6, and A2), and occipital (O1 and O2) regions	No statistically significant results
Palm et al. (2009) [34]	1 66-year-old female MD (major depression) patient	Averaged absolute and relative power in delta, theta, alpha, and beta bands for frontal (Fp1, Fp2, F3, FC1, F4, FC2, FC5, F7, F8, FC6, and Fz), central (T3, T4, CP5, CP6, C3, C4, and Cz), and posterior (T5, T6, P3, P4, Pz, O1, and O2) regions	Decreased absolute power in delta band in frontal area and decreased absolute power in alpha band in frontal and central areas. Decreased relative power in delta and theta bands in frontal area and in alpha band in frontal and central areas post tES treatment
Wang et al. (2022) [132]	24 PSEI (post-stroke executive impairment) patients, 7 female, mean age 54.08 ± 10.53	Averaged absolute power in delta, theta, alpha, and beta bands in left prefrontal (Fp1, AF3, F3, and F7), left central (C3), left occipital (O1), right prefrontal (Fp2, AF4, F4, and F8), right central (C4), right occipital (O2), prefrontal (Fp1, AF3, F3, F7, Fp2, AF4, F4, F8, and Fz), central (C3, C4, and Cz), and occipital (O1, O2, and Oz) regions	Higher theta-band absolute power after stimulation in the left central region than before stimulation
Maimon et al. (2022) [136]	6 DOC patients, 1 female, range (24–81 y.o.)	Frontal MMN N1 peak amplitudes, frontal theta VC9 biomarker activity, and mean prefrontal theta-band power	Two patients with significant differences between standard-tone N1 amplitudes and deviant-tone N1 amplitudes before tDCS treatment, and three patients exhibited a significant MMN post tDCS treatment. Absolute frontal theta power increased in 4 patients and decreased in 1. VC9 activity significantly increased in 3 patients and decreased in 1
Emonson et al. (2019) [104]	20 younger adults, 10 female (mean age 24.50 ± 4.48); 20 older adults, 11 female (mean age 65.47 ± 5.62); 9 MCI patients, 4 female (mean age 72.11 ± 5.75)	For TEP at rest: P30/N40, P60, N100, and P200 in F1, FZ, and F2. ERP analysis for 2-back task: N100, P150, N250, and P300 in posterior and frontal regions	In the young, P30 and P60 reduced post-tES amplitude and N250 increased post-tES amplitude; in the elderly, N250 increased post-tES amplitude
Rassovsky et al. (2018) [92]	38 schizophrenia patients, 32% females, mean age 42.7 ± 8.57, range (23–55 y.o.)	MMN in Fz using a passive-attention auditory duration deviant paradigm; P300 in Pz using an active attention auditory oddball paradigm. N170 in P7 and P8 during another task	No statistically significant results
Murphy et al. (2023) [150]	49 MDD patients, 29 females, mean age = 28.46 ± 6.12, range (18–65 y.o.)	Event-Related Synchronization (ERS%) and Event-Related Desynchronization (ERD%) within the theta, upper alpha, and gamma frequency bands in all acquisition channels	Increase in upper-alpha ERS% in parieto-occipital regions 5 min post tES and in left frontal and lateral parieto-occipital regions 25 min post tES. tDCS > sham in both conditions
Hoy et al. (2015) [55]	18 schizophrenia patients, 6 females, mean age 42.17 ± 11.04	ERS% for correct trials only in the gamma band during the active interval and the reference interval in F3.	Significant ERS% increase in gamma band 40 min post stimulation 2 mA for tES. Significant decrease in gamma at 40 min post stimulation for sham tES.
O’Neil-Pirozzi et al. (2017) [80]	4 Neurotypical patients, one male, mean age = 51.6, range (44–59 y.o.); 4 TBI, two males, mean age = 43, range (35–53 y.o.)	P300 in Cz and absolute power in theta and alpha bands from each electrode in frontal, parietal, and occipital areas	Increased P300 amplitude after anodal stimulation compared to sham only in TBI
Ulam et al. (2015) [60]	26 TBI patients, 4 females, mean age = 33.52 ± 12.25	Relative power Z scores in delta, theta, alpha, beta, and high beta bands at 6 different time points in F3 (anode) and Fp2 (cathode)	Active tDCS group had greater delta at Fp2 than the sham group for EEG#1 and EEG#2. Greater delta at F3 for the active tDCS group compared to the sham group at EEG#3. Greater total delta for the active tDCS group at EEG#2 and #3 compared to the sham group in Fp2. Significant decrease in theta between EEG#2 and EEG#3 for the active tDCS group in F3. Significant decrease in delta between EEG#1 and #6 for the active group in F3 and Fp2. Significant increase in alpha from EEG#1 to #6 for the active group in F3 and in Fp2. Significant difference at EEG#6 with greater alpha relative power in F3 and Fp2 for active vs. sham group

Another scarcely used parameter is ERS%, which has bee nanalyzed by both Murphy et al. (2023) and Hoy et al. (2015) [55,150]. In both studies, Event-Related Synchronization (ERS%) was specifically evaluated at the F3 electrode, although within broader analyses. Murphy et al. (2023) [150] assessed ERS% and ERD% across multiple frequency bands, including the theta, upper alpha, and gamma bands across all recorded channels, while Hoy et al. (2015) [55] focused more narrowly on gamma ERS% during correction trials at F3. Despite examining ERS% at the same channel location, the two studies reported different significant effects. Murphy et al. (2023) [150] found an increase in upper alpha ERS% in parieto-occipital regions 5 min after stimulation and, later, increases in the left frontal and lateral parieto-occipital areas 25 min after stimulation. In contrast, Hoy et al. (2015) [55] observed a significant increase in gamma ERS% at F3 40 min following 2 mA stimulation, along with a significant decrease in gamma ERS% in the sham condition at the same time point.

These results underscore how far the field remains from identifying generalizable EEG effects of specific tES setups. Progress in this direction may depend on the adoption of standardized stimulation protocols and homogeneous EEG feature extraction methods to evaluate stimulation outcomes. These observations highlight the need for greater standardization in channel selection and feature computation to enable more robust cross-study comparisons. Furthermore, the presence of non-significant findings in some studies emphasizes the need for additional research to clarify the neurophysiological impact of left frontal stimulation [66,92]. Notably, even when stimulation parameters are fixed, statistically significant outcomes are not consistently observed across participants.

### 3.6. Distribution of Participant Categories

**Figure 3:**
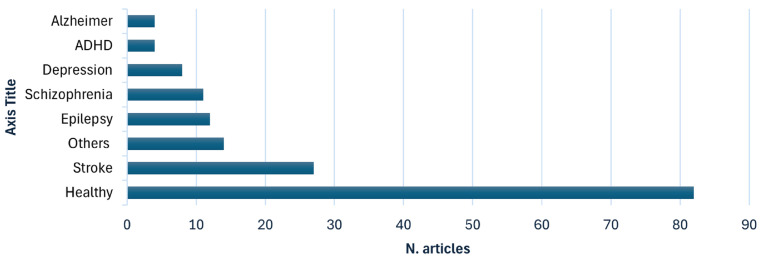
Bar chart showing the number of articles by population: healthy participants and patient groups by pathology; articles may include more than one population type.

Analysis of the sample distribution, as shown in Figure 3, reveals a predominance of studies conducted in clinical populations, accounting for 51% of the total, while the remaining 49% involves healthy subjects. In particular, patients affected by stroke represent the second largest group, at 16%, indicating strong interest in neuromodulation for post-stroke rehabilitation. Epilepsy (7%) and schizophrenia (8%) follow, with promising outcomes in modulating dysfunctional cortical activity. Patients diagnosed with depression account for 6%, while those with Attention-Deficit/Hyperactivity Disorder (ADHD) and Alzheimer’s Disease (AD) make up 2% each. Lastly, 8% falls under the “Others” category, each with an incidence below 2%, covering conditions such as dementia, affective disorder, fibromyalgia, and burnout syndrome. Across different pathological conditions, studies adopt various stimulation setups based on literature indicating how each disorder affects specific brain areas, often identified through EEG features. In studies involving healthy subjects, stimulation typically targets the prefrontal cortex, reflecting a focus on cognitive processes, particularly memory-related functions.

### 3.7. Distribution of Current Waveform

**Figure 4:**
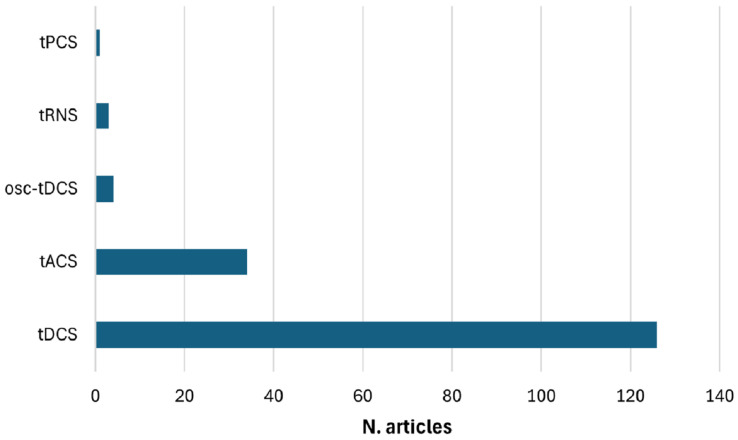
Bar chart illustrating the number of current waveforms applied across studies. Articles may include more than one current waveform. Expanded acronyms are reported in the Abbreviations section.

Analysis of the collected data indicates tDCS as the most frequently employed type of stimulation, accounting for approximately 74% of cases, as reported in Figure 4. This predominance results from its relative technical simplicity, allowing for use over many years, with the development of documents and guidelines supporting applications in healthcare, which are also supported by consistent preliminary outcomes across clinical and cognitive domains. The literature has provided substantial evidence supporting its effectiveness in modulating cortical activity, facilitating widespread use in both experimental and therapeutic contexts [178]. In tDCS, anodal stimulation is now well established to increase neuronal excitability through a depolarizing shift in membrane potential, facilitating action potential initiation [179]. In contrast, cathodal stimulation induces hyperpolarization, leading to inhibition of action potential initiation [180]. tACS emerges as the second most utilized approach, with a prevalence of 20%. Despite its lower adoption compared to tDCS, tACS is gaining interest due to its ability to selectively influence neural oscillatory activity at physiologically relevant frequencies. Its more limited application is related to reduced standardization and the increased complexity of protocols, requiring an additional parameter—namely, stimulation frequency—to produce appropriate spectral shifts toward greater balance. Less conventional modalities, including tRNS (2%), tPCS (1%), and osc-tDCS (2%), show minimal usage. This low prevalence may result from their still-exploratory nature, limited protocol validation, and a lack of robust evidence of clinical efficacy.

The evident imbalance in usage across techniques suggests a need for broader methodological exploration and increased openness to alternative experimental protocols. The predominance of tDCS reflects its perceived effectiveness but also limits comprehensive understanding of the potential benefits offered by alternative approaches such as tPCS and tRNS.

### 3.8. Distribution of Anode and Cathode Positions

**Figure 5:**
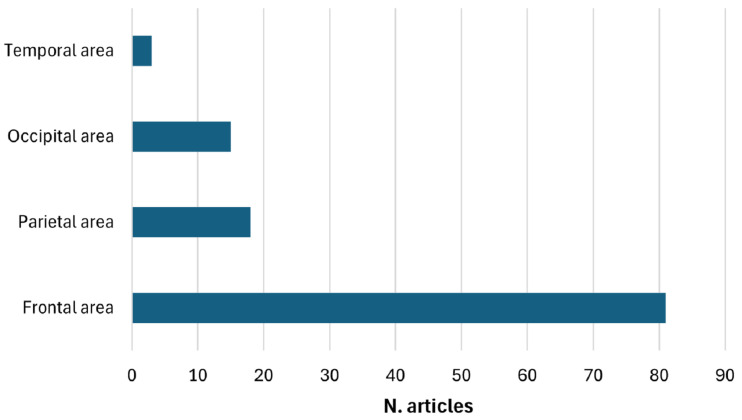
Bar chart illustrating the number of anode placements across studies. Articles may include more than one anode placement.

The literature analysis revealed a marked predominance in the use of specific cortical sites for the placement of the anode electrode during tES. As shown in Figure 5, more than half of the reviewed studies (70%) positioned the anodal electrode over the frontal area. This finding reflects the growing interest in the role of the frontal lobe, particularly the Dorsolateral Prefrontal Cortex (DLPFC), in cognitive functions including attention, working memory, and emotional regulation [181]. The high frequency of stimulation in this area suggests continued preference for the targeting of the frontal cortex in investigations of cognitive and therapeutic effects of tES.

The parietal and the occipital areas were also commonly targeted, accounting for 15% and 12% of studies, respectively. Parietal stimulation is often associated with research on spatial attention, multisensory integration, and body awareness [182], while occipital stimulation is related to attention or visual functions. In contrast, the temporal lobe appeared in only 3% of the reviewed articles, indicating a significantly lower usage. However, given the temporal lobe’s involvement in auditory processing, language, and episodic memory, its relevance may increase as tES applications expand into these cognitive domains [183]. Overall, the distribution of stimulation sites highlights a clear trend toward frontal and motor areas, likely due to stronger empirical support, anatomical accessibility, and the availability of established protocols. Nevertheless, a broader exploration of less frequently targeted regions remains crucial to fully characterize the neuromodulatory potential of tES across both clinical and experimental contexts.

**Figure 6:**
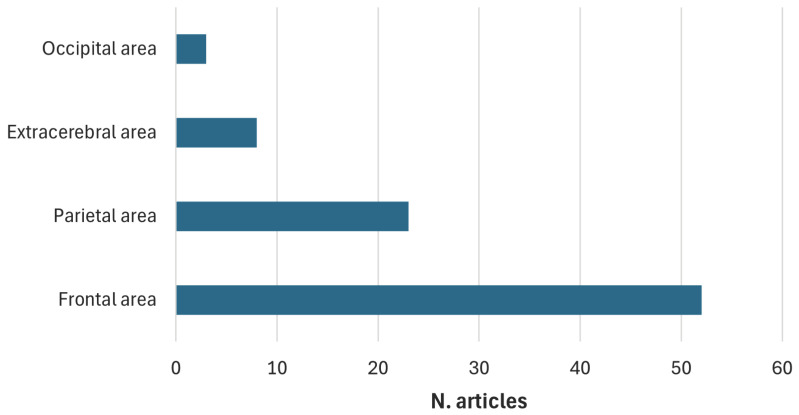
Bar chart illustrating the number of cathode placements across studies. Articles may include more than one cathode placement.

The distribution of cathode placements across studies reveals consistent trends in methodological choices for tES protocols. In most cases, as illustrated in Figure 6, the cathode is positioned over cortical area—specifically, over the frontal region in 61% of studies, the parietal region in 26%, and the occipital region in 3%. The remaining 10% of studies opt for extracerebral placements, including sites such as the shoulder or mastoid. In these cases, the cathode serves as a reference electrode. This strategy aims to minimize unintended cortical effects from the reference and to isolate stimulation effects at the active site [184].

## 4. Discussion

This systematic review provides an updated synthesis of current practices and challenges in the integration of EEG with transcranial electrical stimulation (tES). Four key findings emerge in response to the predefined research questions. First (RQ-I), only a limited number of studies (3%) employed EEG solely to design stimulation parameters, such as electrode placement or stimulation frequency, using individual neurophysiological markers (e.g., peak alpha frequency [23] or functional connectivity [22]). Second (RQ-II), ten studies (6%) applied real-time EEG-guided modulation of stimulation parameters, positioning closed-loop stimulation as a promising strategy to adapt tES based on ongoing brain activity, despite some limitations still existing. Third (RQ-III), the majority of studies (91%) used EEG to assess the effects of tES. Most analyses focused on spectral power changes in alpha, theta, and beta bands or on event-related potentials (ERPs). Although standardized stimulation configurations are frequently employed, indicating a general convergence in protocol design, the resulting electrophysiological effects remain highly heterogeneous across subjects [53,59,93,104,105]. Moreover, only in a few cases was the relationship between EEG and clinical outcomes analyzed. Fourth (RQ-IV), studies applying identical stimulation protocols (e.g., tDCS with anode over F3 and cathode over Fp2) reported highly variable EEG outcomes. This variability persisted, even when targeting the same EEG features using consistent stimulation settings, and often resulted in non-significant statistical effects [34,66]. Such findings underscore the considerable inter-individual variability of EEG responses to tES.

A non-negligible 10% of the included articles combined both uses of EEG, relying on pre-treatment EEG recordings to define stimulation parameters and subsequently assessing treatment impact. In most of these studies, the EEG baseline condition was found to predict specific responses to stimulation. An analysis of the temporal distribution of the publications included in this review shows that only 30% of the studies were published in the last five years (since 2021). This distribution is likely explained by the COVID-19 pandemic (2020–2022), which led to a temporary slowdown of in-lab experimental research and an increase in theoretical or review papers, inevitably impacting the number of experimental studies involving simultaneous EEG–tES recording. Indeed, a renewed upward trend can be observed after the pandemic. It should also be noted that the single study published in 2025 reflects the fact that the literature search was conducted up to January 2025.

Based on the articles included in this review, four major themes emerge: (i) inter-individual variability, often linked to phenotypic heterogeneity; (ii) inconsistent reporting of stimulation parameters; (iii) the early development of closed-loop EEG tES approaches; and (iv) the limited attention given to the relationship between electroencephalographic effects and clinical outcomes of tES, which is often restricted to descriptive or associative statistical analyses. With respect to variability, most tES studies defined stimulation protocols based solely on clinical diagnosis, without integrating neurophysiological features such as resting-state EEG patterns, age, or sex—all of which can influence current distribution and neural response. A critical limitation to the generalization of EEG findings across patients with the same diagnosis is the phenotypic heterogeneity within clinical populations. While this issue has not yet been thoroughly addressed, the work of Dagnino et al. (2023) [143] offers a compelling demonstration. Using unsupervised clustering on resting-state EEG data collected prior to tES in a healthy pediatric sample, the authors identified distinct EEG phenotypes associated with different behavioral responses to tDCS. This supports the idea that EEG phenotyping may account for inter-individual variability and improve prediction of tES outcomes. Given the increasing attention to transdiagnostic frameworks (e.g., Research Domain Criteria, Hierarchical Taxonomy of Psychopathology), future research could explore the relationship between EEG-derived phenotypes and cross-cutting symptom clusters (e.g., affective dysregulation and dysfunctional arousal), beyond traditional Diagnostic and Statistical Manual of Mental Disorders (DSM) categories. This approach may help explain some of the variability observed so far in tES response, which might be obscured when relying solely on categorical diagnoses.

**Figure 7:**
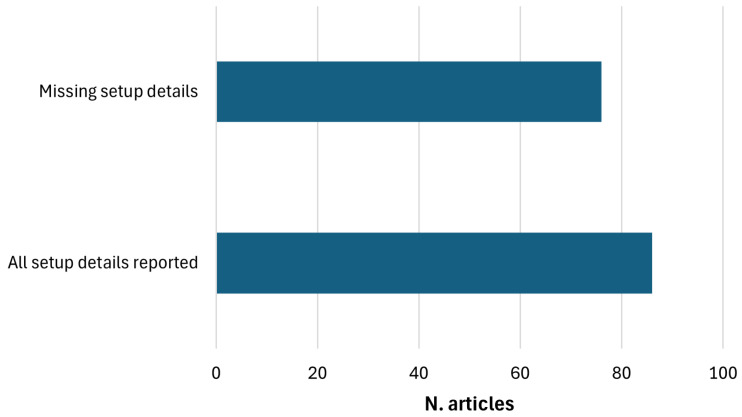
Percentage distribution of articles reporting complete versus incomplete information on tES setup. Incomplete information is considered when at least one of subsequent parameters is missing: stimulation time, current amplitude, electrode material, or electrode size.

**Figure 8:**
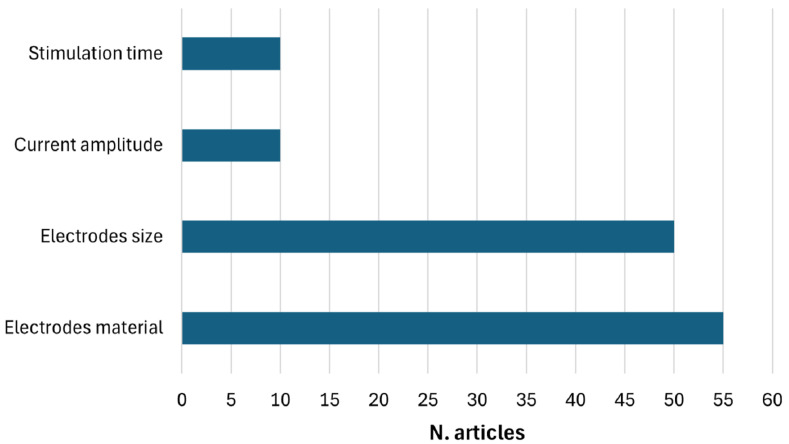
Bar chart showing the number of articles not reporting each parameter of the stimulation setup.

Another critical barrier to progress in EEG–tES research lies in the lack of methodological transparency. As shown in Figure 7, nearly half of the reviewed studies failed to fully report key stimulation parameters, such as electrode material, size, current intensity, and stimulation duration. In a more detailed analysis represented in the bar chart in Figure 8, nearly half of the reviewed studies failed to fully report key stimulation parameters, such as electrode material, size, current intensity, and stimulation duration. The most frequently omitted information concerned the electrode material and size. These omissions compromise the reproducibility of protocols and the interpretability of neurophysiological results, especially considering that variations in electrode characteristics significantly influence the distribution and magnitude of the electric field. For instance, in three studies, only the anode location was reported, without specifying the cathode position [39,42,160], preventing accurate estimation of current density and the overall montage configuration. Incomplete reporting obstructs comparison across studies and limits the generalization of findings. In future work, the development of shared minimum reporting standards for stimulation parameters (e.g., electrode material, intensity, and duration) would be highly valuable to improve methodological reproducibility and facilitate comparison across studies.

Studies proposing adaptive real-time stimulation aim to develop therapeutic interventions in line with the evolution of medicine toward personalized approaches. Most available studies report significant clinical efficacy after stimulation across cognitive and functional domains, including working memory, declarative memory, motor memory, metamemory, sustained attention, visual detection, and reductions in depressive symptoms. Declarative memory exhibits a strong association with SWs, slow and synchronized EEG deflections involved in memory consolidation processes. The up states of SWs during NREM sleep represent phases of maximal cortical excitability and mnemonic reactivation [185]. Delivering stimulation in synchrony with these events increases the probability of strengthening circuits involved in declarative memory and produces selective behavioral effects. Evidence shows improved performance on generalized stimuli but no effect on repeated stimuli, suggesting that closed-loop tACS enhances cognitive integration and schematization of new information rather than mechanical recall of familiar content. These effects correlate with transient modulation of slow wave power and enhanced slow wave–spindle coupling, indicating a direct link between oscillatory modulation and cognitive benefit [24,25]. A closed-loop approach based on SWs has also been proposed, with a focus on metamemory, a metacognitive function reflecting the ability to monitor and evaluate memory quality and closely related to declarative memory. In this context, STAMPs, unique patterns of transcranial electrical stimulation with a specific spatiotemporal distribution of currents, have been used to tag episodic experiences acquired in virtual reality and to selectively reactivate them during sleep. Results show improved metamemory sensitivity [26,27], accompanied by increased theta-band connectivity, greater network efficiency in the spindle band, and beta-band path length changes predictive of metacognitive improvement. On the other hand, temporal coupling with sleep spindles, particularly in the fast range (15–16 Hz), has proven effective in enhancing motor memory reorganization [28]. Motor memory underlies the acquisition and stabilization of motor skills and movement sequences, and spindle activity contributes to brain plasticity and the consolidation of both declarative and procedural memories [186]. Moreover, the magnitude of spindle enhancement predicted motor improvements, with similar correlations also observed on sham nights, providing direct evidence for a causal role of fast sleep spindles in motor memory consolidation. Alpha rhythm also plays a central role in both physiological and pathological contexts. Stabilization of alpha activity enhances cognitive functions and improves performance in tasks involving working memory [29] and visual perception [30]. Real-time monitoring of the individual alpha frequency (IAF) provides a strategy to maximize modulation specificity [31,32]. However, in one study [30], high inter-individual variability in IAF limited alpha power enhancement with closed-loop approaches, producing no significant differences in clinical and neurophysiological outcomes compared with sham. Furthermore, recent work [33] introduced entropy-based metrics including approximate entropy, sample entropy, fuzzy entropy, and multiscale extensions. These measures capture nonlinear EEG dynamics and provide insight into cognitive states, with higher values typically reflecting concentration and lower values indicating relaxation or fatigue. For this reason, such metrics have been employed to investigate the effect of tDCS on sustained attention. In the only patient-based study [31], increased alpha oscillations (8–12 Hz) in the prefrontal cortex represent a pathological correlate of MDD. Delivering stimulation exclusively during this state produced significant reductions in alpha power closely associated with improvements in depressive symptoms [31]. This finding illustrates the potential of clinically relevant EEG biomarkers to serve as a robust anchor for stimulation and to enhance the effectiveness of closed-loop approaches. Although closed-loop tES appears promising for the adaptation of stimulation to ongoing EEG dynamics in real time, current studies present limitations. Firstly, most studies recruited healthy participants, limiting generalization to clinical populations. Additionally, only two studies performed a direct and exhaustive comparison between fixed and adaptive stimulation, reaching opposite conclusions. In particular, Stecher et al. (2021) [30] reported greater efficacy of fixed stimulation for the improvement of visual detection, whereas Caravati et al. (2024) [33] demonstrated superior effects of adaptive stimulation in enhancing sustained attention. The remaining literature contrasted closed-loop protocols only with the sham, limiting the possibility of attributing observed improvements to specific neurophysiological mechanisms driven by real-time adjustment of stimulation parameters.

Furthermore, current literature is defined by the lack of correlation analyses linking neurophysiological changes induced by tES to clinical or cognitive outcomes. In several cases, EEG was recorded pre- and post intervention, but these changes were not statistically modeled alongside behavioral data. Integrating EEG features, stimulation parameters, and clinical outcomes into unified statistical frameworks could provide a more comprehensive understanding of tES efficacy. In conclusion, the integration of EEG and tES within a precision medicine framework remains in its early stages. To unlock the full therapeutic potential of tES, future studies should prioritize (i) EEG-based phenotypic stratification, (ii) standardized and transparent reporting of stimulation protocols, (iii) the identification of functional relationships between EEG and clinical outcomes, and (iv) the implementation of real-time closed-loop EEG–tES systems. Addressing these gaps will be crucial for the advancement of both the understanding of the mechanisms of action of tES and its clinical efficacy. Finally, to improve interdisciplinary usability, future or complementary publications might consider including brief interpretive notes or summary tables linking EEG patterns to known psychological functions (e.g., increased beta → increased arousal; decreased delta → reduced arousal). This would support clinicians, therapists, and psychologists in translating EEG findings into functional insights. A limitation of this review is the inclusion of studies published only in English. This language restriction, while ensuring accurate interpretation of methodological details, may have led to the exclusion of relevant contributions in other languages, thereby potentially under-representing some research conducted in non-English-speaking countries. Moreover, this review included only studies involving human participants. While animal models provide valuable insights into fundamental neurophysiological mechanisms, substantial anatomical and physiological differences (e.g., head structure, tissue conductivity, cortical folding, and brain size) limit the direct translation of tES findings to humans. This choice aimed to ensure methodological homogeneity and clinical relevance in the synthesis of results.

## 5. Conclusions

This systematic review examines the combined use of EEG and tES in both clinical and healthy populations, focusing on EEG’s role in protocol design (RQ-I), monitoring (RQ-II), and assessment (RQ-III), as well as the generalizability of EEG responses to specific tES configurations (RQ-IV). A systematic search across Google Scholar, PubMed, Scopus, IEEE Xplore, ScienceDirect, and Web of Science identified 162 articles and abstracts later evaluated in relation to the four research questions.

Most of the included studies applied EEG post stimulation to assess neurophysiological effects (RQ-III), and ten implemented real-time feedback for dynamic adjustment of stimulation parameters (RQ-II), while only five used EEG to guide protocol design (RQ-I). Heterogeneity in stimulation setups, the analyzed EEG features, and participant characteristics hindered cross-study comparisons and the identification of generalizable EEG responses (RQ-IV). Despite the promise of theoretical frameworks, the majority of studies rely on standardized electrode placements and neglect inter-individual neurophysiological variability. EEG is mostly employed for post hoc assessment rather than protocol customization. Closed-loop approaches address this gap by providing a promising means to adapt stimulation in real time through EEG monitoring, predominantly investigated in healthy subjects. However, they still present some limitations, including the absence of direct comparisons with fixed tES protocols. Moreover, the lack of consistent reporting on stimulation parameters and poor methodological standardization reduce reproducibility and limit the clinical translation of findings. Future research should prioritize real-time EEG-tES integration, transparent protocol documentation, and the identification of the functional relationship between EEG and clinical outcomes. Such efforts will be essential to advance toward adaptive, phenotype-informed neuromodulation strategies. A further improvement could involve the inclusion of ecological and functional outcome measures (e.g., self-regulation, daily functioning, and quality of life) in order to extend the impact of the EEG–tES model to clinically meaningful dimensions observable in real-world settings.

## Data Availability

Data sharing is not applicable.

## Abbreviations

The following abbreviations are used in this manuscript:

**Abbreviation**	**Meaning**
**Clinical Case**	
MDD	Major Depressive Disorder
ADHD	Attention-Deficit Hyperactivity Disorder
ASD	Autism Spectrum Disorder
PD	Parkinson’s Disease
AD	Alzheimer’s Disease
OUD	Opioid Use Disorder
DOC/DoC	Disorder of Consciousness
TBI	Traumatic Brain Injury
PTSD	Post-Traumatic Stress Disorder
MCS	Minimally Conscious State
MCI	Mild Cognitive Impairment
OCD	Obsessive Compulsive Disorder
UWS	Unresponsive Wakefulness Syndrome
**Stimulation electrode position**	
A position	Anode position
C position	Cathode position
DLPFC	Dorsolateral Prefrontal Cortex
PFC	Prefrontal Cortex
rVLPFC	Ventrolateral Prefrontal Cortex
vmPFC	Ventromedial Prefrontal Cortex
M1	Primary Motor Cortex
PPC	Posterior Parietal Cortex
TPJ	Temporal–Parietal Junction
EF	Epileptogenic Focus
**EEG feature**	
ERP	Event-Related Potential
RP	Relative Power
TP	Total Power
ERSP	Event-Related Spectral Perturbation
ERS/ERD	Event-Related Synchronization/Desynchronization
PSD	Power Spectral Density
GMFP/LMFP	Global/Local Mean Field Power
ApEn	Approximate Entropy
C-ApEn	Approximate Cross Entropy
SaEn	Sample Entropy
FuEn	Fuzzy Entropy
MSEI	Multiscale Sample Entropy Index
MFEI	Multiscale Fuzzy Entropy Index
TEP	Transcranial Evoked Potentials
MEP	Motor Evoked Potentials
SSVEP/VEP	Steady-State Visual Evoked Potential/Visual Evoked Potentials
MMN	Mismatch Negativity
EFR	Envelope Following Response
PLV	Phase-Locking Value
PAF	Peak Alpha Frequency
LPC	Late Positive Component
LP	Late Potential
DMN	Default Mode Network
MER	Maximum Entropy Ratio
PRI	Power-Ratio Index
pdBSI	Pairwise-Derived Brain Symmetry Index
rBSI	Revised Brain Symmetry Index
MSS	Mean State Shift
SV	State Variance
LPP	Late Positive Potential
PAP	Peak Alpha Power
EF	Epileptogenic Focus
IAF	Individual Alpha Frequency
ITF	Individual Theta Frequency
EDs	Epileptiform Discharges
LORETA	Low-Resolution Brain Electromagnetic Tomography
PLI	Phase Lag Index
**Stimulation Type**	
tDCS	Transcranial Direct Current Stimulation
tACS	Transcranial Alternating Current Stimulation
tRNS	Transcranial Random Noise Stimulation
so-tDCS	Slow Oscillatory Transcranial Direct Current Stimulation
HD-tDCS	High-Definition Transcranial Direct Current Stimulation
HD-tACS	High-Definition Transcranial Alternating Current Stimulation
tPCS	Transcranial Pulsed Current Stimulation
hf-tRNS	High-Frequency Transcranial Random Noise Stimulation
osc-tDCS	Oscillatory Transcranial Direct Current Stimulation
**Data Analysis Method**	
S	Statistical analysis
ML	Machine Learning
DL	Deep Learning

## References

[1] Ekhtiari H., Tavakoli H., Addolorato G., Baeken C., Bonci A., Campanella S., Castelo-Branco L., Challet-Bouju G., Clark V.P., Claus E. (2019). Transcranial electrical and magnetic stimulation (tES and TMS) for addiction medicine: A consensus paper on the present state of the science and the road ahead. Neurosci. Biobehav. Rev..

[2] Fertonani A., Miniussi C. (2017). Transcranial electrical stimulation: What we know and do not know about mechanisms. Neuroscientist.

[3] Bikson M., Esmaeilpour Z., Adair D., Kronberg G., Tyler W.J., Antal A., Datta A., Sabel B.A., Nitsche M.A., Loo C. (2019). Transcranial electrical stimulation nomenclature. Brain Stimul..

[4] Lefaucheur J.P., Antal A., Ayache S.S., Benninger D.H., Brunelin J., Cogiamanian F., Cotelli M., De Ridder D., Ferrucci R., Langguth B. (2017). Evidence-based guidelines on the therapeutic use of transcranial direct current stimulation (tDCS). Clin. Neurophysiol..

[5] Antal A., Alekseichuk I., Bikson M., Brockmöller J., Brunoni A.R., Chen R., Cohen L., Dowthwaite G., Ellrich J., Flöel A. (2017). Low intensity transcranial electric stimulation: Safety, ethical, legal regulatory and application guidelines. Clin. Neurophysiol..

[6] Miranda P., Cox C.D., Alexander M., Danev S., Lakey J.R. (2019). Overview of current diagnostic, prognostic, and therapeutic use of EEG and EEG-based markers of cognition, mental, and brain health. Integr. Mol. Med..

[7] Choi J., Kwon M., Jun S.C. (2020). A systematic review of closed-loop feedback techniques in sleep studies—Related issues and future directions. Sensors.

[8] Ruffini G., Modolo J., Sanchez-Todo R., Salvador R., Santarnecchi E. (2020). Clinical drivers for personalization of transcranial current stimulation (tES 3.0). Non Invasive Brain Stimulation in Psychiatry and Clinical Neurosciences.

[9] Beumer S., Boon P., Klooster D.C., van Ee R., Carrette E., Paulides M.M., Mestrom R.M. (2022). Personalized tdcs for focal epilepsy—A narrative review: A data-driven workflow based on imaging and eeg data. Brain Sci..

[10] Simula S., Daoud M., Ruffini G., Biagi M.C., Benar C.G., Benquet P., Wendling F., Bartolomei F. (2022). Transcranial current stimulation in epilepsy: A systematic review of the fundamental and clinical aspects. Front. Neurosci..

[11] Yang D., Shin Y.I., Hong K.S. (2021). Systemic review on transcranial electrical stimulation parameters and EEG/fNIRS features for brain diseases. Front. Neurosci..

[12] Riva J.J., Malik K.M., Burnie S.J., Endicott A.R., Busse J.W. (2012). What is your research question? An introduction to the PICOT format for clinicians. J. Can. Chiropr. Assoc..

[13] Liberati A., Altman D.G., Tetzlaff J., Mulrow C., Gøtzsche P.C., Ioannidis J.P., Clarke M., Devereaux P.J., Kleijnen J., Moher D. (2009). The PRISMA statement for reporting systematic reviews and meta-analyses of studies that evaluate healthcare interventions: Explanation and elaboration. BMJ.

[14] Kitchenham B. (2004). Procedures for Performing Systematic Reviews.

[15] Mateen F., OH J., Tergas A., Bhayani N., Kamdar B. Title-abstract versus title-only citation screening strategies for systematic reviews and meta-analyses. Proceedings of the Cochrane Colloquium Abstracts.

[16] National Collaborating Centre for Methods and Tools (2010). Quality Assessment Tool for Quantitative Studies.

[17] Perestelo-Pérez L. (2013). Standards on how to develop and report systematic reviews in Psychology and Health. Int. J. Clin. Health Psychol..

[18] Rethlefsen M.L., Kirtley S., Waffenschmidt S., Ayala A.P., Moher D., Page M.J., Koffel J.B. (2021). PRISMA-S: An extension to the PRISMA statement for reporting literature searches in systematic reviews. Syst. Rev..

[19] Fregni F., Thome-Souza S., Nitsche M.A., Freedman S.D., Valente K.D., Pascual-Leone A. (2006). A controlled clinical trial of cathodal DC polarization in patients with refractory epilepsy. Epilepsia.

[20] San-Juan D., Sarmiento C.I., Hernandez-Ruiz A., Elizondo-Zepeda E., Santos-Vázquez G., Reyes-Acevedo G., Zúñiga-Gazcón H., Zamora-Jarquín C.M. (2016). Transcranial alternating current stimulation: A potential risk for genetic generalized epilepsy patients (study case). Front. Neurol..

[21] Andrade S.M., da Silva-Sauer L., de Carvalho C.D., de Araújo E.L.M., Lima E.d.O., Fernandes F.M.L., Moreira K.L.d.A.F., Camilo M.E., Andrade L.M.M.d.S., Borges D.T. (2023). Identifying biomarkers for tDCS treatment response in Alzheimer’s disease patients: A machine learning approach using resting-state EEG classification. Front. Hum. Neurosci..

[22] De Ridder D., Vanneste S. (2012). EEG driven tDCS versus bifrontal tDCS for tinnitus. Front. Psychiatry.

[23] Mokhtarinejad E., Tavakoli M., Ghaderi A.H. (2024). Exploring the correlation and causation between alpha oscillations and one-second time perception through EEG and tACS. Sci. Rep..

[24] Ketz N., Jones A.P., Bryant N.B., Clark V.P., Pilly P.K. (2018). Closed-loop slow-wave tACS improves sleep-dependent long-term memory generalization by modulating endogenous oscillations. J. Neurosci..

[25] Jones A.P., Choe J., Bryant N.B., Robinson C.S., Ketz N.A., Skorheim S.W., Combs A., Lamphere M.L., Robert B., Gill H.A. (2018). Dose-dependent effects of closed-loop tACS delivered during slow-wave oscillations on memory consolidation. Front. Neurosci..

[26] Hubbard R.J., Zadeh I., Jones A.P., Robert B., Bryant N.B., Clark V.P., Pilly P.K. (2021). Brain connectivity alterations during sleep by closed-loop transcranial neurostimulation predict metamemory sensitivity. Netw. Neurosci..

[27] Pilly P.K., Skorheim S.W., Hubbard R.J., Ketz N.A., Roach S.M., Lerner I., Jones A.P., Robert B., Bryant N.B., Hartholt A. (2020). One-shot tagging during wake and cueing during sleep with spatiotemporal patterns of transcranial electrical stimulation can boost long-term metamemory of individual episodes in humans. Front. Neurosci..

[28] Lustenberger C., Boyle M.R., Alagapan S., Mellin J.M., Vaughn B.V., Fröhlich F. (2016). Feedback-controlled transcranial alternating current stimulation reveals a functional role of sleep spindles in motor memory consolidation. Curr. Biol..

[29] Haslacher D., Cavallo A., Reber P., Kattein A., Thiele M., Nasr K., Hashemi K., Sokoliuk R., Thut G., Soekadar S.R. (2024). Working memory enhancement using real-time phase-tuned transcranial alternating current stimulation. Brain Stimul..

[30] Stecher H.I., Notbohm A., Kasten F.H., Herrmann C.S. (2021). A comparison of closed loop vs. fixed frequency tACS on modulating brain oscillations and visual detection. Front. Hum. Neurosci..

[31] Schwippel T., Pupillo F., Feldman Z., Walker C., Townsend L., Rubinow D., Frohlich F. (2024). Closed-loop transcranial alternating current stimulation for the treatment of major depressive disorder: An open-label pilot study. Am. J. Psychiatry.

[32] Zarubin G., Gundlach C., Nikulin V., Villringer A., Bogdan M. (2020). Transient amplitude modulation of alpha-band oscillations by short-time intermittent closed-loop tACS. Front. Hum. Neurosci..

[33] Caravati E., Barbeni F., Chiarion G., Raggi M., Mesin L. (2024). Closed-Loop Transcranial Electrical Neurostimulation for Sustained Attention Enhancement: A Pilot Study towards Personalized Intervention Strategies. Bioengineering.

[34] Palm U., Keeser D., Schiller C., Fintescu Z., Reisinger E., Baghai T.C., Mulert C., Padberg F. (2009). Transcranial direct current stimulation in a patient with therapy-resistant major depression. World J. Biol. Psychiatry.

[35] Zaehle T., Sandmann P., Thorne J.D., Jäncke L., Herrmann C.S. (2011). Transcranial direct current stimulation of the prefrontal cortex modulates working memory performance: Combined behavioural and electrophysiological evidence. BMC Neurosci..

[36] Zaehle T., Beretta M., Jäncke L., Herrmann C.S., Sandmann P. (2011). Excitability changes induced in the human auditory cortex by transcranial direct current stimulation: Direct electrophysiological evidence. Exp. Brain Res..

[37] Kasashima-Shindo Y., Fujiwara T., Ushiba J., Matsushika Y., Kamatani D., Oto M., Ono T., Nishimoto A., Shindo K., Kawakami M. (2015). Brain-computer interface training combined with transcranial direct current stimulation in patients with chronic severe hemiparesis: Proof of concept study. J. Rehabil. Med..

[38] Kongthong N., Minami T., Nakauchi S. (2013). Semantic processing in subliminal face stimuli: An EEG and tDCS study. Neurosci. Lett..

[39] Rütsche B., Hauser T., Jäncke L., Grabner R. (2013). P 56. Modulating arithmetic performance: A tDCS/EEG study. Clin. Neurophysiol..

[40] Lazarev V., Tamborino T., Bikson M., Ferreira M., deAzevedo L., Caparelli-Dáquer E. (2013). P 235. Focal EEG effects of High Definition tDCS (HD-tDCS) detected by EEG photic driving. Clin. Neurophysiol..

[41] Mangia A.L., Pirini M., Cappello A. (2014). Transcranial direct current stimulation and power spectral parameters: A tDCS/EEG co-registration study. Front. Hum. Neurosci..

[42] Lauro L.J.R., Rosanova M., Mattavelli G., Convento S., Pisoni A., Opitz A., Bolognini N., Vallar G. (2014). TDCS increases cortical excitability: Direct evidence from TMS–EEG. Cortex.

[43] Roy A., Baxter B., He B. (2014). High-definition transcranial direct current stimulation induces both acute and persistent changes in broadband cortical synchronization: A simultaneous tDCS–EEG study. IEEE Trans. Biomed. Eng..

[44] Crivelli D., Canavesio Y., Pala F., Finocchiaro R., Cobelli C., Lecci G., Balconi M. (2014). Neuromodulation (tDCS) effect on executive functions in healthy aging: Clinical and EEG evidences. Neuropsychol. Trends.

[45] von Mengden I., Garcia C., Glos M., Schöbel C., Fietze I., Penzel T. (2014). Influence of Slow Oscillating Transcranial Direct Current Stimulation (so-tDCS) on Sleep EEG with focus on Spindle Density and Cognitive Performance on Healthy Subjects. Clin. Neurophysiol..

[46] Powell T.Y., Boonstra T.W., Martin D.M., Loo C.K., Breakspear M. (2014). Modulation of cortical activity by transcranial direct current stimulation in patients with affective disorder. PLoS ONE.

[47] Dominguez A., Socas R., Marrero H., Leon N., LLabres J., Enriquez E. (2014). Transcranial direct current stimulation improves word production in conduction aphasia: Electroencephalographic and behavioral evidences. Int. J. Clin. Health Psychol..

[48] Miller J., Berger B., Sauseng P. (2015). Anodal transcranial direct current stimulation (tDCS) increases frontal–midline theta activity in the human EEG: A preliminary investigation of non-invasive stimulation. Neurosci. Lett..

[49] D’Atri A., De Simoni E., Gorgoni M., Ferrara M., Ferlazzo F., Rossini P.M., De Gennaro L. (2015). Frequency-dependent effects of oscillatory-tDCS on EEG oscillations: A study with better oscillation detection method (BOSC). Arch. Ital. Biol..

[50] Jindal U., Sood M., Chowdhury S.R., Das A., Kondziella D., Dutta A. Corticospinal excitability changes to anodal tDCS elucidated with NIRS-EEG joint-imaging: An ischemic stroke study. Proceedings of the 2015 37th Annual International Conference of the IEEE Engineering in Medicine and Biology Society (EMBC).

[51] Sood M., Jindal U., Chowdhury S.R., Das A., Kondziella D., Dutta A. Anterior temporal artery tap to identify systemic interference using short-separation NIRS measurements: A NIRS/EEG-tDCS study. Proceedings of the 2015 37th Annual International Conference of the IEEE Engineering in Medicine and Biology Society (EMBC).

[52] Amatachaya A., Jensen M.P., Patjanasoontorn N., Auvichayapat N., Suphakunpinyo C., Janjarasjitt S., Ngernyam N., Aree-uea B., Auvichayapat P. (2015). The short-term effects of transcranial direct current stimulation on electroencephalography in children with autism: A randomized crossover controlled trial. Behav. Neurol..

[53] Cosmo C., Ferreira C., Miranda J.G.V., Do Rosario R.S., Baptista A.F., Montoya P., De Sena E.P. (2015). Spreading effect of tDCS in individuals with attention-deficit/hyperactivity disorder as shown by functional cortical networks: A randomized, double-blind, sham-controlled trial. Front. Psychiatry.

[54] Del Felice A., Magalini A., Masiero S. (2015). Slow-oscillatory transcranial direct current stimulation modulates memory in temporal lobe epilepsy by altering sleep spindle generators: A possible rehabilitation tool. Brain Stimul..

[55] Hoy K.E., Bailey N.W., Arnold S.L., Fitzgerald P.B. (2015). The effect of transcranial direct current stimulation on gamma activity and working memory in schizophrenia. Psychiatry Res..

[56] Dutta A., Jacob A., Chowdhury S.R., Das A., Nitsche M.A. (2015). EEG-NIRS based assessment of neurovascular coupling during anodal transcranial direct current stimulation-a stroke case series. J. Med. Syst..

[57] Wu D., Wang J., Yuan Y. (2015). Effects of transcranial direct current stimulation on naming and cortical excitability in stroke patients with aphasia. Neurosci. Lett..

[58] Jindal U., Sood M., Dutta A., Chowdhury S.R. (2015). Development of point of care testing device for neurovascular coupling from simultaneous recording of EEG and NIRS during anodal transcranial direct current stimulation. IEEE J. Transl. Eng. Health Med..

[59] Ang K.K., Guan C., Phua K.S., Wang C., Zhao L., Teo W.P., Chen C., Ng Y.S., Chew E. (2015). Facilitating effects of transcranial direct current stimulation on motor imagery brain-computer interface with robotic feedback for stroke rehabilitation. Arch. Phys. Med. Rehabil..

[60] Ulam F., Shelton C., Richards L., Davis L., Hunter B., Fregni F., Higgins K. (2015). Cumulative effects of transcranial direct current stimulation on EEG oscillations and attention/working memory during subacute neurorehabilitation of traumatic brain injury. Clin. Neurophysiol..

[61] Impey D., Knott V. (2015). Effect of transcranial direct current stimulation (tDCS) on MMN-indexed auditory discrimination: A pilot study. J. Neural Transm..

[62] Sood M., Besson P., Muthalib M., Jindal U., Perrey S., Dutta A., Hayashibe M. (2016). NIRS-EEG joint imaging during transcranial direct current stimulation: Online parameter estimation with an autoregressive model. J. Neurosci. Methods.

[63] Cappon D., Goljahani A., Laera G., Bisiacchi P. (2016). Interactions between non invasive transcranial brain stimulation (tACS) and brain oscillations: A quantitative EEG study. Int. J. Psychophysiol..

[64] Caldiroli C.L., Balconi M. (2015). The effect of tDCS on EEG profile during a semantic motor task divided in a correct and incorrect ways. Proceedings of the International Symposium on Pervasive Computing Paradigms for Mental Health.

[65] Marceglia S., Mrakic-Sposta S., Rosa M., Ferrucci R., Mameli F., Vergari M., Arlotti M., Ruggiero F., Scarpini E., Galimberti D. (2016). Transcranial direct current stimulation modulates cortical neuronal activity in Alzheimer’s disease. Front. Neurosci..

[66] Liu A., Bryant A., Jefferson A., Friedman D., Minhas P., Barnard S., Barr W., Thesen T., O’Connor M., Shafi M. (2016). Exploring the efficacy of a 5-day course of transcranial direct current stimulation (TDCS) on depression and memory function in patients with well-controlled temporal lobe epilepsy. Epilepsy Behav..

[67] Dunn W., Rassovsky Y., Wynn J.K., Wu A.D., Iacoboni M., Hellemann G., Green M.F. (2016). Modulation of neurophysiological auditory processing measures by bilateral transcranial direct current stimulation in schizophrenia. Schizophr. Res..

[68] D’Agata F., Peila E., Cicerale A., Caglio M.M., Caroppo P., Vighetti S., Piedimonte A., Minuto A., Campagnoli M., Salatino A. (2016). Cognitive and neurophysiological effects of non-invasive brain stimulation in stroke patients after motor rehabilitation. Front. Behav. Neurosci..

[69] Ashikhmin A., Shishelova A., Aliev R. (2017). tDCS provokes sustainable changes in EEG and reorganizes autonomic modulation of heart rate. Brain Stimul. Basic Transl. Clin. Res. Neuromodulation.

[70] Angulo-Sherman I.N., Rodríguez-Ugarte M., Sciacca N., Iáñez E., Azorín J.M. (2017). Effect of tDCS stimulation of motor cortex and cerebellum on EEG classification of motor imagery and sensorimotor band power. J. Neuroeng. Rehabil..

[71] Angulo-Sherman I.N., Rodríguez-Ugarte M., Iáñez E., Ortíz M., Azorín J.M. Effect on the classification of motor imagery in EEG after applying anodal tDCS with a 4 × 1 ring montage over the motor cortex. Proceedings of the 2017 International Conference on Rehabilitation Robotics (ICORR).

[72] Grande G., Golemme M., Tatti E., Chiesa S., Van Velzen J., Luft C.D.B., Cappelletti M. (2017). P127 A combined EEG and alpha tACS study on visual working memory in healthy ageing. Clin. Neurophysiol..

[73] Donaldson P., Kirkovski M., Rinehart N., Enticott P. (2017). Social cognition and the temporoparietal junction: A double-blind HD-tDCS EEG study. Brain Stimul. Basic Transl. Clin. Res. Neuromodulation.

[74] Berger A., Pixa N., Doppelmayr M. (2017). Frequency-specific after-effects of transcranial alternating current stimulation (tACS) on motor learning: Preliminary data of a simultaneous tACS-EEG-NIRS study. Brain Stimul. Basic Transl. Clin. Res. Neuromodulation.

[75] Cortes M., Edwards D., Putrino D. (2017). Anodal tDCS decreases total EEG power at rest and alters brain signaling during fatigue in high performance athletes. Brain Stimul. Basic Transl. Clin. Res. Neuromodulation.

[76] Ladenbauer J., Ladenbauer J., Külzow N., de Boor R., Avramova E., Grittner U., Flöel A. (2017). Promoting sleep oscillations and their functional coupling by transcranial stimulation enhances memory consolidation in mild cognitive impairment. J. Neurosci..

[77] Impey D., Baddeley A., Nelson R., Labelle A., Knott V. (2017). Effects of transcranial direct current stimulation on the auditory mismatch negativity response and working memory performance in schizophrenia: A pilot study. J. Neural Transm..

[78] Naros G., Gharabaghi A. (2017). Physiological and behavioral effects of *β*-tACS on brain self-regulation in chronic stroke. Brain Stimul..

[79] Yuan Y., Wang J., Wu D., Huang X., Song W. (2017). Effect of transcranial direct current stimulation on swallowing apraxia and cortical excitability in stroke patients. Top. Stroke Rehabil..

[80] O’Neil-Pirozzi T.M., Doruk D., Thomson J.M., Fregni F. (2017). Immediate memory and electrophysiologic effects of prefrontal cortex transcranial direct current stimulation on neurotypical individuals and individuals with chronic traumatic brain injury: A pilot study. Int. J. Neurosci..

[81] Boudewyn M., Roberts B.M., Mizrak E., Ranganath C., Carter C.S. (2019). Prefrontal transcranial direct current stimulation (tDCS) enhances behavioral and EEG markers of proactive control. Cogn. Neurosci..

[82] Kang J., Cai E., Han J., Tong Z., Li X., Sokhadze E.M., Casanova M.F., Ouyang G., Li X. (2018). Transcranial direct current stimulation (tDCS) can modulate EEG complexity of children with autism spectrum disorder. Front. Neurosci..

[83] Mane R., Chew E., Phua K.S., Ang K.K., Vinod A.P., Guan C. Quantitative EEG as biomarkers for the monitoring of post-stroke motor recovery in BCI and tDCS rehabilitation. Proceedings of the 2018 40th Annual International Conference of the IEEE Engineering in Medicine and Biology Society (EMBC).

[84] Cukic M., Stokic M., Radenkovic S., Ljubisavljevic M., Pokrajac D.D. (2018). The shift in brain-state induced by tDCS: An EEG study. arXiv.

[85] Friedrich J., Beste C. (2018). Paradoxical, causal effects of sensory gain modulation on motor inhibitory control—A tDCS, EEG-source localization study. Sci. Rep..

[86] Mondini V., Mangia A.L., Cappello A. (2018). Single-session tDCS over the dominant hemisphere affects contralateral spectral EEG power, but does not enhance neurofeedback-guided event-related desynchronization of the non-dominant hemisphere’s sensorimotor rhythm. PLoS ONE.

[87] Holgado D., Zandonai T., Hopker J., Zabala M., Ciria L., Sanabria D. (2018). Null effects of tDCS over the Left Prefrontal Cortex on Self-paced Exercise and EEG. J. Sci. Cycl..

[88] Berger A., Pixa N.H., Steinberg F., Doppelmayr M. (2018). Brain oscillatory and hemodynamic activity in a bimanual coordination task following transcranial alternating current stimulation (tACS): A combined EEG-fNIRS study. Front. Behav. Neurosci..

[89] Ferrucci R., Mrakic-Sposta S., Gardini S., Ruggiero F., Vergari M., Mameli F., Arighi A., Spallazzi M., Barocco F., Michelini G. (2018). Behavioral and neurophysiological effects of transcranial direct current stimulation (tDCS) in fronto-temporal dementia. Front. Behav. Neurosci..

[90] Shahsavar Y., Ghoshuni M., Talaei A. (2018). Quantifying clinical improvements in patients with depression under the treatment of transcranial direct current stimulation using event related potentials. Australas. Phys. Eng. Sci. Med..

[91] Meiron O., Gale R., Namestnic J., Bennet-Back O., David J., Gebodh N., Adair D., Esmaeilpour Z., Bikson M. (2018). High-Definition transcranial direct current stimulation in early onset epileptic encephalopathy: A case study. Brain Inj..

[92] Rassovsky Y., Dunn W., Wynn J.K., Wu A.D., Iacoboni M., Hellemann G., Green M.F. (2018). Single transcranial direct current stimulation in schizophrenia: Randomized, cross-over study of neurocognition, social cognition, ERPs, and side effects. PLoS ONE.

[93] Hordacre B., Moezzi B., Ridding M.C. (2018). Neuroplasticity and network connectivity of the motor cortex following stroke: A transcranial direct current stimulation study. Hum. Brain Mapp..

[94] Nicolo P., Magnin C., Pedrazzini E., Plomp G., Mottaz A., Schnider A., Guggisberg A.G. (2018). Comparison of neuroplastic responses to cathodal transcranial direct current stimulation and continuous theta burst stimulation in subacute stroke. Arch. Phys. Med. Rehabil..

[95] Straudi S., Bonsangue V., Mele S., Craighero L., Montis A., Fregni F., Lavezzi S., Basaglia N. (2019). Bilateral M1 anodal transcranial direct current stimulation in post traumatic chronic minimally conscious state: A pilot EEG-tDCS study. Brain Inj..

[96] D’Atri A., Scarpelli S., Gorgoni M., Alfonsi V., Annarumma L., Giannini A.M., Ferrara M., Ferlazzo F., Rossini P.M., De Gennaro L. (2019). Bilateral theta transcranial alternating current stimulation (tACS) modulates EEG activity: When tACS works awake it also works asleep. Nat. Sci. Sleep.

[97] Dondé C., Brevet-Aeby C., Poulet E., Mondino M., Brunelin J. (2019). Potential impact of bifrontal transcranial random noise stimulation (tRNS) on the semantic Stroop effect and its resting-state EEG correlates. Neurophysiol. Clin..

[98] Donaldson P.H., Kirkovski M., Rinehart N.J., Enticott P.G. (2019). A double-blind HD-tDCS/EEG study examining right temporoparietal junction involvement in facial emotion processing. Soc. Neurosci..

[99] Dowsett J., Herrmann C.S., Dieterich M., Taylor P.C. (2020). Shift in lateralization during illusory self-motion: EEG responses to visual flicker at 10 Hz and frequency-specific modulation by tACS. Eur. J. Neurosci..

[100] Bueno-Lopez A., Eggert T., Dorn H., Danker-Hopfe H. (2019). Slow oscillatory transcranial direct current stimulation (so-tDCS) during slow wave sleep has no effects on declarative memory in healthy young subjects. Brain Stimul..

[101] Handiru V.S., Guan C., Ang K.K., Chew E. (2019). Abstract# 130: EEG Beta-band Coherence for Prognosis of Motor Recovery in Stroke Patients with tDCS-BCI Intervention. Brain Stimul. Basic Transl. Clin. Res. Neuromodulation.

[102] Willms M., Brucar L., Muller A., Vila-Rodrigues F., Rosenblatt C., Babul N.V. (2019). Exploring tDCS-induced changes in EEG power and network connectivity in youth concussion: Preliminary findings. Brain Stimul. Basic Transl. Clin. Res. Neuromodulation.

[103] Mastakouri A.A., Schölkopf B., Grosse-Wentrup M. Beta power may meditate the effect of Gamma-TACS on motor performance. Proceedings of the 2019 41st Annual International Conference of the IEEE Engineering in Medicine and Biology Society (EMBC).

[104] Emonson M., Fitzgerald P., Rogasch N., Hoy K. (2019). Neurobiological effects of transcranial direct current stimulation in younger adults, older adults and mild cognitive impairment. Neuropsychologia.

[105] Cespón J., Rodella C., Miniussi C., Pellicciari M. (2019). Behavioural and electrophysiological modulations induced by transcranial direct current stimulation in healthy elderly and Alzheimer’s disease patients: A pilot study. Clin. Neurophysiol..

[106] Alexander M.L., Alagapan S., Lugo C.E., Mellin J.M., Lustenberger C., Rubinow D.R., Fröhlich F. (2019). Double-blind, randomized pilot clinical trial targeting alpha oscillations with transcranial alternating current stimulation (tACS) for the treatment of major depressive disorder (MDD). Transl. Psychiatry.

[107] Meiron O., Gale R., Namestnic J., Bennet-Back O., Gebodh N., Esmaeilpour Z., Mandzhiyev V., Bikson M. (2019). Antiepileptic effects of a novel non-invasive neuromodulation treatment in a subject with early-onset epileptic encephalopathy: Case report with 20 sessions of HD-tDCS intervention. Front. Neurosci..

[108] Ahn S., Mellin J.M., Alagapan S., Alexander M.L., Gilmore J.H., Jarskog L.F., Fröhlich F. (2019). Targeting reduced neural oscillations in patients with schizophrenia by transcranial alternating current stimulation. Neuroimage.

[109] Singh A., Trapp N.T., De Corte B., Cao S., Kingyon J., Boes A.D., Parker K.L. (2019). Cerebellar theta frequency transcranial pulsed stimulation increases frontal theta oscillations in patients with schizophrenia. Cerebellum.

[110] Schoellmann A., Scholten M., Wasserka B., Govindan R.B., Krüger R., Gharabaghi A., Plewnia C., Weiss D. (2019). Anodal tDCS modulates cortical activity and synchronization in Parkinson’s disease depending on motor processing. NeuroImage Clin..

[111] Mane R., Chew E., Phua K.S., Ang K.K., Robinson N., Vinod A., Guan C. (2019). Prognostic and monitory EEG-biomarkers for BCI upper-limb stroke rehabilitation. IEEE Trans. Neural Syst. Rehabil. Eng..

[112] Bao S.C., Wong W.W., Leung T.W.H., Tong K.Y. (2018). Cortico-muscular coherence modulated by high-definition transcranial direct current stimulation in people with chronic stroke. IEEE Trans. Neural Syst. Rehabil. Eng..

[113] Luna F.G., Román-Caballero R., Barttfeld P., Lupiáñez J., Martín-Arévalo E. (2020). A High-Definition tDCS and EEG study on attention and vigilance: Brain stimulation mitigates the executive but not the arousal vigilance decrement. Neuropsychologia.

[114] El-Hagrassy M., Duarte D., Lu J., Uygur-Kucukseymen E., Münger M., Thibaut A., Lv P., Morales-Quezada L., Fregni F. (2021). EEG modulation by different transcranial direct current stimulation (tDCS) montages: A randomized double-blind sham-control mechanistic pilot trial in healthy participants. Expert Rev. Med. Devices.

[115] de Melo G.A., de Oliveira E.A., dos Santos Andrade S.M.M., Fernández-Calvo B., Torro N. (2020). Comparison of two tDCS protocols on pain and EEG alpha-2 oscillations in women with fibromyalgia. Sci. Rep..

[116] Sergiou C.S., Santarnecchi E., Romanella S.M., Wieser M.J., Franken I.H.A., Rassin E., van Dongen J.D.M. (2020). tDCS Targeting the Ventromedial Prefrontal Cortex Reduces Reactive Aggression and Modulates Electrophysiological Responses: A HD-tDCS/EEG Randomized Controlled Trial in a Forensic Population [Pre-Registration]. OSF. https://osf.io/cjgdt/.

[117] Pross B., Siamouli M., Pogarell O., Falkai P., Hasan A., Strube W. (2018). S177. Frontal cortical plasticity in schizophrenia patients examined by LTP-inducing anodal TDCS and repetitive EEG. Schizophr. Bull..

[118] Gangemi A., Colombo B., Fabio R.A. (2021). Effects of short-and long-term neurostimulation (tDCS) on Alzheimer’s disease patients: Two randomized studies. Aging Clin. Exp. Res..

[119] Nikolin S., Martin D., Loo C.K., Iacoviello B.M., Boonstra T.W. (2020). Assessing neurophysiological changes associated with combined transcranial direct current stimulation and cognitive-emotional training for treatment-resistant depression. Eur. J. Neurosci..

[120] Breitling C., Zaehle T., Dannhauer M., Tegelbeckers J., Flechtner H.H., Krauel K. (2020). Comparison between conventional and HD-tDCS of the right inferior frontal gyrus in children and adolescents with ADHD. Clin. Neurophysiol..

[121] Boudewyn M.A., Scangos K., Ranganath C., Carter C.S. (2020). Using prefrontal transcranial direct current stimulation (tDCS) to enhance proactive cognitive control in schizophrenia. Neuropsychopharmacology.

[122] Jahshan C., Wynn J.K., Roach B.J., Mathalon D.H., Green M.F. (2020). Effects of Transcranial Direct Current Stimulation on Visual Neuroplasticity in Schizophrenia. Clin. EEG Neurosci..

[123] Zhang X., Liu B., Li N., Li Y., Hou J., Duan G., Wu D. (2020). Transcranial direct current stimulation over prefrontal areas improves psychomotor inhibition state in patients with traumatic brain injury: A pilot study. Front. Neurosci..

[124] Grasso P.A., Tonolli E., Bortoletto M., Miniussi C. (2021). tDCS over posterior parietal cortex increases cortical excitability but decreases learning: An ERPs and TMS-EEG study. Brain Res..

[125] Zakaria H., Valentine O., Mayza A. (2021). Analysis of quantitative EEG (QEEG) parameters on the effect of transcranial direct current stimulation (TDCS) on post-stroke patients. AIP Conf. Proc..

[126] Ghin F., O’Hare L., Pavan A. (2021). Electrophysiological aftereffects of high-frequency transcranial random noise stimulation (hf-tRNS): An EEG investigation. Exp. Brain Res..

[127] Mostafavi H., Dadashi M., Faridi A., Kazemzadeh F., Eskandari Z. (2022). Using bilateral tDCS to modulate EEG amplitude and coherence of men with opioid use disorder under methadone therapy: A sham-controlled clinical trial. Clin. EEG Neurosci..

[128] Mai G., Howell P. (2020). Causal relationship between the right auditory cortex and speech-evoked frequency-following response: Evidence from combined tDCS and EEG. bioRxiv.

[129] Wang C., Zhang Y., Chen Y., Song P., Yu H., Sun C., Du J. (2021). Comparison and Affecting Factors of Three tDCS Montages in Motor Recovery of Chronic Stroke Patients: A Resting-State EEG Study [Preprint]. Research Square. https://www.researchgate.net/publication/348261758_Comparison_and_Affecting_Factors_of_Three_tDCS_Montages_in_Motor_Recovery_of_Chronic_Stroke_Patients_A_Resting-State_EEG_Study.

[130] Hu P., He Y., Liu X., Ren Z., Liu S. Modulating emotion processing using transcranial alternating current stimulation (tACS)-A sham-controlled study in healthy human participants. Proceedings of the 2021 43rd Annual International Conference of the IEEE Engineering in Medicine & Biology Society (EMBC).

[131] Ghafoor U., Yang D., Hong K.S. (2021). Neuromodulatory effects of HD-tACS/tDCS on the prefrontal cortex: A resting-state fNIRS-EEG study. IEEE J. Biomed. Health Inform..

[132] Wang C., Chen Y., Song P., Yu H., Du J., Zhang Y., Sun C. (2022). Varied response of EEG rhythm to different tDCS protocols and lesion hemispheres in stroke subjects with upper limb dysfunction. Neural Plast..

[133] Liu B., Zhang X., Li Y., Duan G., Hou J., Zhao J., Guo T., Wu D. (2022). tDCS-EEG for predicting outcome in patients with unresponsive wakefulness syndrome. Front. Neurosci..

[134] Kim S., Yang C., Dong S.Y., Lee S.H. (2022). Predictions of tDCS treatment response in PTSD patients using EEG based classification. Front. Psychiatry.

[135] Westwood S.J., Bozhilova N., Criaud M., Lam S.L., Lukito S., Wallace-Hanlon S., Kowalczyk O.S., Kostara A., Mathew J., Wexler B.E. (2022). The effect of transcranial direct current stimulation (tDCS) combined with cognitive training on EEG spectral power in adolescent boys with ADHD: A double-blind, randomized, sham-controlled trial. IBRO Neurosci. Rep..

[136] Maimon N.B., Molcho L., Jaul E., Intrator N., Barron J., Meiron O. EEG reactivity changes captured via mobile BCI device following tDCS intervention—A pilot-study in disorders of consciousness (DOC) patients. Proceedings of the 2022 10th International Winter Conference on Brain-Computer Interface (BCI).

[137] Ayub M., Ullah K., Khan M.J., Farooq H., Khan A. Cognitive Improvement Estimation using EEG Imaging after tDCS Therapy. Proceedings of the 2022 International Conference on Emerging Trends in Electrical, Control, and Telecommunication Engineering (ETECTE).

[138] Palmisano A., Tatti E., Pezanko L., Cappon D., Macome J., Koch G., Smeralda C., Rivolta D., El Fakhri G., Pascual-Leone A. (2022). PC016/# 697 PERTURBATION-BASED TACS-EEG BIOMARKERS OF GAMMA ACTIVITY IN ALZHEIMER’S DISEASE: E-POSTER VIEWING. Neuromodulation.

[139] Cheng J., Li P., Tang Y., Zhang C., Lin L., Gao J., Wang Z. (2022). Transcranial direct current stimulation improve symptoms and modulates cortical inhibition in obsessive-compulsive disorder: A TMS-EEG study. J. Affect. Disord..

[140] de Souza Moura B., Hu X.S., DosSantos M.F., DaSilva A.F. (2022). Study protocol of tDCS based pain modulation in head and neck cancer patients under chemoradiation therapy condition: An fNIRS-EEG study. Front. Mol. Neurosci..

[141] Mosayebi-Samani M., Agboada D., Mutanen T.P., Haueisen J., Kuo M.F., Nitsche M.A. (2023). Transferability of cathodal tDCS effects from the primary motor to the prefrontal cortex: A multimodal TMS-EEG study. Brain Stimul..

[142] Yeh T.C., Huang C.C.Y., Chung Y.A., Park S.Y., Im J.J., Lin Y.Y., Ma C.C., Tzeng N.S., Chang H.A. (2023). Online left-hemispheric in-phase frontoparietal theta tACS modulates theta-band EEG source-based large-scale functional network connectivity in patients with schizophrenia: A randomized, double-blind, sham-controlled clinical trial. Biomedicines.

[143] Dagnino P.C., Braboszcz C., Kroupi E., Splittgerber M., Brauer H., Dempfle A., Breitling-Ziegler C., Prehn-Kristensen A., Krauel K., Siniatchkin M. (2023). Stratification of responses to tDCS intervention in a healthy pediatric population based on resting-state EEG profiles. Sci. Rep..

[144] Sergiou C.S., Tatti E., Romanella S.M., Santarnecchi E., Weidema A.D., Rassin E.G., Franken I.H., van Dongen J.D. (2023). The effect of HD-tDCS on brain oscillations and frontal synchronicity during resting-state EEG in violent offenders with a substance dependence. Int. J. Clin. Health Psychol..

[145] Kim S., Yang C., Dong S.Y., Lee S.H. (2023). Deep Convolutional Neural Network based tDCS Prognosis Classification in PTSD Patients using EEG Spectrograms. Brain Stimul. Basic Transl. Clin. Res. Neuromodulation.

[146] Roy S., Fan Y., Nitsche M. (2023). ASSESSING THE ROLE OF TRANSCRANIAL DIRECT CURRENT STIMULATION (TDCS) IN RESCUING STRESS-INDUCED WORKING MEMORY (WM) DEFICITS–AN EEG-BASED STUDY. IBRO Neurosci. Rep..

[147] Liu M., Xu G., Yu H., Wang C., Sun C., Guo L. (2022). Effects of transcranial direct current stimulation on EEG power and brain functional network in stroke patients. IEEE Trans. Neural Syst. Rehabil. Eng..

[148] Fabio R.A., Suriano R., Gangemi A. (2024). Effects of Transcranial Direct Current Stimulation on Potential P300-Related Events and Alpha and Beta EEG Band Rhythms in Parkinson’s Disease. J. Integr. Neurosci..

[149] Chan M.M., Choi C.X., Tsoi T.C., Shea C.K., Yiu K.W., Han Y.M. (2023). Effects of multisession cathodal transcranial direct current stimulation with cognitive training on sociocognitive functioning and brain dynamics in autism: A double-blind, sham-controlled, randomized EEG study. Brain Stimul..

[150] Murphy O., Hoy K., Wong D., Bailey N., Fitzgerald P., Segrave R. (2023). Effects of transcranial direct current stimulation and transcranial random noise stimulation on working memory and task-related EEG in major depressive disorder. Brain Cogn..

[151] Wang X., Ouyang J., Kang A., Wang L., Zhang J., Yan T., Zhang J., Yan Z. Gamma tACS Reshapes Low-Dimensional Trajectories of Brain Activity in Working Memory. Proceedings of the 2023 17th International Conference on Complex Medical Engineering (CME).

[152] Wang Y., Liu W., Wang Y., Ouyang G., Guo Y. (2024). Long-term HD-tDCS modulates dynamic changes of brain activity on patients with disorders of consciousness: A resting-state EEG study. Comput. Biol. Med..

[153] Tarantino V., Fontana M.L., Buttà A., Ficile S., Oliveri M., Mandalà G., Smirni D. (2024). Increase in EEG Alpha-to-theta Ratio After transcranial Direct Current Stimulation (tDCS) in Patients with Disorders of Consciousness: A Pilot Study. NeuroRehabilitation.

[154] Vimolratana O., Aneksan B., Siripornpanich V., Hiengkaew V., Prathum T., Jeungprasopsuk W., Khaokhiew T., Vachalathiti R., Klomjai W. (2024). Effects of anodal tDCS on resting state eeg power and motor function in acute stroke: A randomized controlled trial. J. Neuroeng. Rehabil..

[155] Singh V., Verma R., Shriyam S., Gandhi T.K. (2024). Evaluating tDCS Intervention Effectiveness via Functional Connectivity Network on Resting-State EEG Data in Major Depressive Disorder. arXiv.

[156] Couto T.A., Gao F., Lak D.C., Yuan Z. (2024). Combined EEG-tDCS approach in resting state to reduce comorbid anxiety and depressive symptoms in affective disorders: A sham-controlled pilot study. IBRO Neurosci. Rep..

[157] Liu C.L., Su K.H., Horng Y.S., Chen C.L., Huang S.H., Wu C.Y. (2024). Theory-driven EEG indexes for tracking motor recovery and predicting the effects of hybridizing tDCS with mirror therapy in stroke patients. IEEE Trans. Neural Syst. Rehabil. Eng..

[158] Wynn S.C., Marshall T.R., Nyhus E. (2025). Utilizing tACS to enhance memory confidence and EEG to predict individual differences in brain stimulation efficacy. Imaging Neurosci..

[159] Yeh T.C., Lin Y.Y., Tzeng N.S., Kao Y.C., Chung Y.A., Chang C.C., Fang H.W., Chang H.A. (2025). Effects of online high-definition transcranial direct current stimulation over left dorsolateral prefrontal cortex on predominant negative symptoms and EEG functional connectivity in patients with schizophrenia: A randomized, double-blind, controlled trial. Psychiatry Clin. Neurosci..

[160] Zhou Y., Zhai H., Wei H. (2024). Acute Effects of Transcranial Direct Current Stimulation Combined with High-Load Resistance Exercises on Repetitive Vertical Jump Performance and EEG Characteristics in Healthy Men. Life.

[161] Zhang S., Cui H., Li Y., Chen X., Gao X., Guan C. (2024). Improving SSVEP-BCI performance through repetitive anodal tDCS-based neuromodulation: Insights from fractal EEG and brain functional connectivity. IEEE Trans. Neural Syst. Rehabil. Eng..

[162] Xiao W., Moncy J.C., Ghazi-Noori A.R., Woodham R.D., Rezaei H., Bramon E., Ritter P., Bauer M., Young A.H., Fu C.H. (2025). Enhanced network synchronization connectivity following transcranial direct current stimulation (tDCS) in bipolar depression: Effects on EEG oscillations and deep learning-based predictors of clinical remission. J. Affect. Disord..

[163] Rocha K., Marinho V., Magalhães F., Carvalho V., Fernandes T., Ayres M., Crespo E., Velasques B., Ribeiro P., Cagy M. (2020). Unskilled shooters improve both accuracy and grouping shot having as reference skilled shooters cortical area: An EEG and tDCS study. Physiol. Behav..

[164] Aktürk T., de Graaf T.A., Güntekin B., Hanoğlu L., Sack A.T. (2022). Enhancing memory capacity by experimentally slowing theta frequency oscillations using combined EEG-tACS. Sci. Rep..

[165] Faria P., Fregni F., Sebastião F., Dias A.I., Leal A. (2012). Feasibility of focal transcranial DC polarization with simultaneous EEG recording: Preliminary assessment in healthy subjects and human epilepsy. Epilepsy Behav..

[166] Auvichayapat N., Rotenberg A., Gersner R., Ngodklang S., Tiamkao S., Tassaneeyakul W., Auvichayapat P. (2013). Transcranial direct current stimulation for treatment of refractory childhood focal epilepsy. Brain Stimul..

[167] Lin L.C., Ouyang C.S., Chiang C.T., Yang R.C., Wu R.C., Wu H.C. (2018). Cumulative effect of transcranial direct current stimulation in patients with partial refractory epilepsy and its association with phase lag index-A preliminary study. Epilepsy Behav..

[168] Tecchio F., Cottone C., Porcaro C., Cancelli A., Di Lazzaro V., Assenza G. (2018). Brain functional connectivity changes after transcranial direct current stimulation in epileptic patients. Front. Neural Circuits.

[169] Dallmer-Zerbe I., Popp F., Lam A.P., Philipsen A., Herrmann C.S. (2020). Transcranial alternating current stimulation (tACS) as a tool to modulate P300 amplitude in attention deficit hyperactivity disorder (ADHD): Preliminary findings. Brain Topogr..

[170] Zaehle T., Rach S., Herrmann C.S. (2010). Transcranial alternating current stimulation enhances individual alpha activity in human EEG. PLoS ONE.

[171] Stecher H.I., Pollok T.M., Strüber D., Sobotka F., Herrmann C.S. (2017). Ten minutes of *α*-tACS and ambient illumination independently modulate EEG *α*-power. Front. Hum. Neurosci..

[172] Khayyer Z., Ngaosuvan L., Sikström S., Ghaderi A.H. (2018). Transcranial direct current stimulation based on quantitative electroencephalogram combining positive psychotherapy for major depression. J. Integr. Neurosci..

[173] Beatrice P.D.K., Guay S., Proulx-Bégin L., Masse I., Lina J.M., Carrier J., De Beaumont L. (2019). Abstract# 129: Characterization of tACS parameters to optimize the increase of EEG alpha power. Brain Stimul. Basic Transl. Clin. Res. Neuromodulation.

[174] Del Felice A., Castiglia L., Formaggio E., Cattelan M., Scarpa B., Manganotti P., Tenconi E., Masiero S. (2019). Personalized transcranial alternating current stimulation (tACS) and physical therapy to treat motor and cognitive symptoms in Parkinson’s disease: A randomized cross-over trial. NeuroImage Clin..

[175] Radecke J.O., Fiene M., Misselhorn J., Herrmann C.S., Engel A.K., Wolters C.H., Schneider T.R. (2023). Personalized alpha-tACS targeting left posterior parietal cortex modulates visuo-spatial attention and posterior evoked EEG activity. Brain Stimul..

[176] Góral-Półrola J., Kochańska E., Cielebąk K., Pąchalska M. (2024). A NEW, NEUROMARKER-BASED, FORM OF COMBINED NEUROFEED-BACK EEG/TDCS TRAINING IN THE REDUCTION OF OCCUPATIONAL BURNOUT SYNDROME IN AN ANAESTHETIC NURSE WORKING WITH COVID-19 PATIENTS. Acta Neuropsychol..

[177] Kim Y., Lee J.H., Yun S., Yang J., Park J.C., Kwon J., Seo J., Min B.K. Enhanced inhibitory control after out-of-phase theta tACS between the lDLPFC and dACC. Proceedings of the 2024 12th International Winter Conference on Brain-Computer Interface (BCI).

[178] Sarkis R.A., Kaur N., Camprodon J.A. (2014). Transcranial direct current stimulation (tDCS): Modulation of executive function in health and disease. Curr. Behav. Neurosci. Rep..

[179] Nitsche M.A., Paulus W. (2000). Excitability changes induced in the human motor cortex by weak transcranial direct current stimulation. J. Physiol..

[180] Nitsche M.A., Nitsche M.S., Klein C.C., Tergau F., Rothwell J.C., Paulus W. (2003). Level of action of cathodal DC polarisation induced inhibition of the human motor cortex. Clin. Neurophysiol..

[181] White L.K., Makhoul W., Teferi M., Sheline Y.I., Balderston N.L. (2023). The role of dlPFC laterality in the expression and regulation of anxiety. Neuropharmacology.

[182] Pearson H.J. (2009). Present and Accounted For: Sensory Stimulation and Parietal Neuroplasticity. J. EMDR Pract. Res..

[183] Malkani R.G., Zee P.C. (2020). Brain stimulation for improving sleep and memory. Sleep Med. Clin..

[184] Noetscher G.M., Yanamadala J., Makarov S.N., Pascual-Leone A. (2014). Comparison of cephalic and extracephalic montages for transcranial direct current stimulation—A numerical study. IEEE Trans. Biomed. Eng..

[185] Göldi M., van Poppel E.A.M., Rasch B., Schreiner T. (2019). Increased neuronal signatures of targeted memory reactivation during slow-wave up states. Sci. Rep..

[186] Boutin A., Doyon J. (2020). A sleep spindle framework for motor memory consolidation. Philos. Trans. R. Soc. B.

